# Spatial and Temporal Variations in Indoor Environmental Conditions, Human Occupancy, and Operational Characteristics in a New Hospital Building

**DOI:** 10.1371/journal.pone.0118207

**Published:** 2015-03-02

**Authors:** Tiffanie Ramos, Sandra Dedesko, Jeffrey A. Siegel, Jack A. Gilbert, Brent Stephens

**Affiliations:** 1 Department of Civil, Architectural and Environmental Engineering, Illinois Institute of Technology, Chicago, Illinois, United States of America; 2 Department of Civil Engineering, University of Toronto, Toronto, Ontario, Canada; 3 Argonne National Laboratory, Institute for Genomic and Systems Biology, Argonne, Illinois, United States of America; 4 Department of Ecology and Evolution, University of Chicago, Chicago, Illinois, United States of America; University of Coimbra, PORTUGAL

## Abstract

The dynamics of indoor environmental conditions, human occupancy, and operational characteristics of buildings influence human comfort and indoor environmental quality, including the survival and progression of microbial communities. A suite of continuous, long-term environmental and operational parameters were measured in ten patient rooms and two nurse stations in a new hospital building in Chicago, IL to characterize the indoor environment in which microbial samples were taken for the Hospital Microbiome Project. Measurements included environmental conditions (indoor dry-bulb temperature, relative humidity, humidity ratio, and illuminance) in the patient rooms and nurse stations; differential pressure between the patient rooms and hallways; surrogate measures for human occupancy and activity in the patient rooms using both indoor air CO_2_ concentrations and infrared doorway beam-break counters; and outdoor air fractions in the heating, ventilating, and air-conditioning systems serving the sampled spaces. Measurements were made at 5-minute intervals over consecutive days for nearly one year, providing a total of ∼8×10^6^ data points. Indoor temperature, illuminance, and human occupancy/activity were all weakly correlated between rooms, while relative humidity, humidity ratio, and outdoor air fractions showed strong temporal (seasonal) patterns and strong spatial correlations between rooms. Differential pressure measurements confirmed that all patient rooms were operated at neutral pressure. The patient rooms averaged about 100 combined entrances and exits per day, which suggests they were relatively lightly occupied compared to higher traffic environments (e.g., retail buildings) and more similar to lower traffic office environments. There were also clear differences in several environmental parameters before and after the hospital was occupied with patients and staff. Characterizing and understanding factors that influence these building dynamics is vital for hospital environments, where they can impact patient health and the survival and spread of healthcare associated infections.

## Introduction

Certain building design, environmental, and operational factors are important to characterize in critical building environments, such as hospitals, because they influence occupant comfort and health [1–5], indoor environmental quality [6–11], and the progression, survival, and transmission of microbial pathogens [12–19]. The Hospital Microbiome Project (http://www.hospitalmicrobiome.com) was designed to investigate microbial communities and building environmental factors inside 10 patient rooms and two nearby nurse stations for one year in a new hospital pavilion at the University of Chicago. The measurement campaign occurred immediately before the hospital was occupied (for approximately one month) and after the introduction of patients and hospital staff (for nearly 11 months). While samples were collected for culture-independent sequence-based analysis of bacterial and fungal communities (i.e., 16S rRNA, 18S rRNA, fungal ITS, metagenomics, etc.), a large dataset of building environmental and operational parameters, or *built environment data*, were also collected. This built environment dataset provides an extensive characterization of the indoor parameters that may have influenced human comfort, indoor environmental quality, and the survival and succession of microbial communities within the hospital.

This work details the field equipment, methodology, and spatial and temporal variations in the built environment data to determine whether there were significant differences in the range of built environment parameters within individual rooms, between different rooms, and between floors. Importantly, this study enables us to examine the impact of time of day, season, and the introduction of human occupants on these built environment parameters to determine the potential implications of their variance on human comfort and indoor environmental quality. Additionally, the potential influence of the built environment parameters on the survival and spread of microbial communities can be hypothesized; however comparisons between the actual built environment and microbial datasets will be made once all microbial samples have been analyzed.

## Materials and Methods

The following sections describe the measurement sites and the built environment measurement and analysis methodology in detail.

### Patient Rooms

Five patient rooms and one nurse station were chosen for sampling on each of two floors (the 9^th^ and 10^th^ floors). The 10 patient rooms were nearly identical, single-occupancy, west-facing perimeter adult inpatient rooms, classified as neutral pressure rooms on the mechanical plans. Their floor plans were flipped such that their sinks and bathrooms were installed along shared walls. While all 10 rooms were patient care units, the 10^th^ floor rooms were devoted to oncology patients who typically had longer stays than patients on the 9^th^ floor. Each room had a floor area of approximately 33 m^2^, including a bathroom of 4.6 m^2^. Ceiling heights were 2.9 m, providing a room volume of approximately 97 m^3^. Large windows spanned the width of the west wall of each room, opposite the sole doorway on the east side. Two 1.5 m slot supply air diffusers were located at the ceiling near the windows, spanning the width of the room. The design supply airflow rate was 765 m^3^/hr according to detail drawings and schedule sheets, with minimum flow rates specified as 663 m^3^/hr. The heating, ventilating, and air-conditioning (HVAC) systems conditioned air centrally in shared air handling units (AHUs) and reheat coils at the supply diffusers provided occupant temperature control within individual rooms. A single 0.6 m × 0.6 m return grille was located at the ceiling near the doorway with a design airflow rate of 595 m^3^/hr and a minimum airflow rate of 493 m^3^/hr, according to the schedule sheets. An additional exhaust grille was located in the bathroom with a constant design airflow rate of 170 m^3^/hr.

### Nurse Stations

Two large nurse stations, one located across the hallway from the patient rooms on each floor, were also selected as sampling locations. These nurse stations consisted of several computer workstations and filing cabinets contained within a large oval-shaped two-tiered desk that separated nurses from hospital visitors and patients. The nurse stations did not contain any windows that were directly external to the outdoor environment.

### HVAC Systems

Patient rooms and nurse stations on each floor were served by AHUs located in the two-level mechanical penthouse on the 11^th^ and 12^th^ floors. The 10^th^ floor was served by a single AHU with a design airflow rate of ∼85,000 m^3^/hr, while the 9^th^ floor was served by a combination of four connected ∼85,000 m^3^/hr AHUs (with a total design airflow rate of ∼340,000 m^3^/hr) that also served the 8^th^ floor. These four AHUs were connected at a common return plenum (or mixing box) where recirculated air was mixed with outdoor air, conditioned, filtered, and delivered into a common supply plenum that then split into four individual supply air plenums for distribution via ductwork throughout the hospital. Each AHU had MERV 7 and MERV 13 pre-filters installed before the heating and cooling coils and supply fans, as well as HEPA filtration installed just before the entrance to the supply plenum. Dampers at the outdoor air intakes were automated for economizer control, which adjusted the outdoor air intake flow rate depending primarily on the outdoor air temperature.

### Site Identifications

In order to de-identify patient rooms and nurse stations for purposes of confidentiality, a naming convention was created to refer to rooms on the 9^th^ and 10^th^ floors of the hospital. Room numbers 101–105 refer to consecutive rooms on the 9^th^ floor, with 100 referring to the nurse station on the same floor. Room numbers 201–205 refer to the consecutive rooms on the 10^th^ floor, directly above those on the 9^th^ floor, with 200 referring to the nurse station on the 10^th^ floor. AHU 6 refers to the single air handling unit that served the 10^th^ floor. AHU 11 refers to the combination of four air handling units that combined to serve the 8^th^ and 9^th^ floors.

### Built Environment Data Collection: Measurements

The built environment data collection campaign and corresponding analysis included long-term characterizations of:

- Indoor environmental conditions, including air dry-bulb temperature, relative humidity, humidity ratio (a measure of absolute humidity or the moisture content of air), and illuminance (a measure of incident light) in the 10 patient rooms and two nurse stations.
- Differential pressure between the 10 patient rooms and the hallways.
- Surrogate measures of human occupancy and activity in the 10 patient rooms using both indoor CO_2_ concentrations and infrared (IR) beam-break counters installed at the patient room doorways.
- Outdoor air fractions in the HVAC systems serving the two floors.

Many of these parameters are suggested in both the MIxS-BE package for minimal metadata collection [20] and in a recent compilation of recommendations for built environment data collection for indoor microbial investigations [21]. The selection of measured parameters was also informed by existing literature on human occupancy, environmental conditions, and HVAC characteristics that are known to influence occupant comfort, indoor environmental quality, and microbial communities in buildings, as described in more detail in the discussion section of this work. Each parameter was measured at 5-minute intervals with an assortment of off-the-shelf sensors. Accuracy, ease of data retrieval, aesthetic impact, battery life, and budgetary constraints were all considered when selecting these measurement devices. Measurements were made over the span of approximately one year (January 22, 2013 to January 15, 2014) while project partners took concurrent daily and weekly microbial samples from a variety of surfaces in patient rooms and nurse stations. Patients and staff were introduced when the hospital opened on February 24, 2013.

### Indoor Environmental Conditions

**Fig 1:**
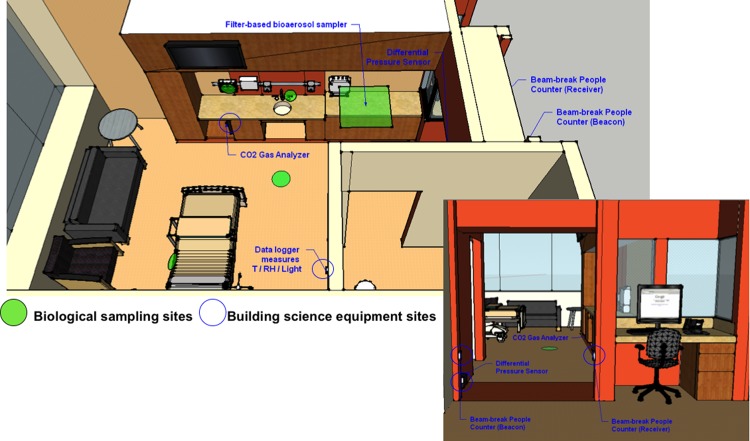
Typical patient room showing locations of built environment sensors and microbial sampling sites. Sketch of a typical patient room in the hospital. Green shaded areas show microbiological sampling sites; areas circled in blue show locations of the building science measurements sites used herein. The nurse stations, although not pictured, were centrally located to the right of the figure (in the gray area).

Onset HOBO U12-012 data loggers were used to record temperature (±0.4°C at 25°C), relative humidity (±2.5% from 10% to 90% RH), and illuminance (uncertainty unknown). In the patient rooms, the data loggers were installed at a height of approximately 1.4 m on the wall adjacent to the patient bed and across from the large windows. This location was chosen because much of the microbial sampling would occur at or around the beds and the data loggers would capture the illuminance representative of the majority of the sampling space. The location of each sensor in the patient rooms is shown in **Fig. 1**. In the two nurse stations, the temperature, relative humidity, and light sensors were mounted vertically on a counter in a central area at approximately the same 1.4 m height (although they are not pictured in **Fig. 1**). The 5-minute measurement interval of these sensors was synchronized with all other Onset HOBO data loggers throughout the hospital. Periodic surface temperature measurements were also made with an IR thermometer on several sampling sites early in the study but were discontinued because they correlated very strongly to indoor air temperatures at the time of measurement.

### Room Pressurization

Onset T-VER-PXU-X differential pressure transducers (±1% of full scale, or ±0.25 Pa) were installed at each patient room doorway and connected to a large battery pack and an Onset HOBO U12 data logger (±2 mV and ± 2.5% of absolute reading) with tubing placed on each side of the doorway to measure the pressure differential between the patient rooms and the adjacent hallway. This measurement was designed to serve as an indicator of whether the rooms were being operated at neutral pressure, positive pressure isolation (i.e., with airflow moving from patient rooms toward the hallway, protecting occupants in patient rooms from airborne hallway interactions), or negative pressure isolation (i.e., with airflow moving from the hallway toward patient rooms, protecting the hallway and other environments outside of the patient room from a particular patient). The pressure sensors required 12 VDC power supply and drew approximately 35 mA when operating. Therefore, the pressure sensors were connected to battery packs with 8 D batteries and housed together in a nondescript black plastic project enclosure box. This box was mounted on the wall with adhesive strips. Clear vinyl tubing was used to measure the pressure differential and data were logged to an Onset HOBO data logger at 5-minute intervals, synchronized with other HOBO loggers throughout the hospital. Field measurements using an Energy Conservatory DG-700 differential pressure sensor confirmed very low, typically neutral pressures with respect to the hallway, so the pressure transducers were set to bi-directional operation with a maximum range of ±25 Pa.

### Human Occupancy and Activity Surrogate Measurements

Patient room occupancy and activity were assessed using two surrogate measurement methods. One was the installation of SenSource PC-TB12-R non-directional, single IR beam-break people counters mounted at a height of approximately 0.6 m at each doorframe to detect the combined number of entrances and exits through the patient room doorways over 5-minute intervals (this was the only measurement device where the 5-minute measurement interval could not be synchronized with all other measurement devices). Dual-direction beam-break counters and other methods exist, but exceeded the budget for this project and/or would violate patient confidentiality concerns. The beam-break measurements in this investigation were most helpful for inferring the level of *activity* through the doorway of each room, or the combined number of entrances and exits over each 5-minute period, but not necessarily the actual time-varying occupancy. However a reasonable estimate of the daily total occupancy in each room could be determined by summing the number of beam-breaks over a 24-hour period and assuming that half of the beam-breaks were entrances and the other half were exits.

To support the IR beam-break measurements, indoor air CO_2_ concentrations were also measured in each patient room using PP Systems SBA-5 CO_2_ gas analyzers (±1% of full-scale, or ∼20 ppm). The CO_2_ concentrations measured in each room were later combined with CO_2_ concentrations measured concurrently in the supply airstream of the HVAC system serving each floor (described in the next section) in order to provide a metric of “room-source” CO_2_ (*C*_*room-source*_ = *C*_*room*_—*C*_*supply*_), or the CO_2_ concentration that results from occupant generation alone, adjusted for varying ventilation rates. These analyzers were installed in another nondescript black plastic project box on a small shelf within the rooms, with clear vinyl tubing running out to the sampled area (at approximately counter height near the edge of the sink). This location was chosen primarily because of easy access to electrical power, since the analyzers required 12 VDC power supplies and could not operate for long durations on batteries. Data from the CO_2_ analyzers were output to another Onset HOBO data logger (±2 mV and ±2.5% of absolute reading) attached to the side of the project box to allow for easy retrieval and for synchronizing with all other HOBO data loggers.

### HVAC System Characterizations and Ventilation Rate Measurements

To assess outdoor air fractions in the HVAC systems serving the sample floors, CO_2_ concentrations were measured in each of the outdoor, return, and supply air streams at each of the two AHUs in the mechanical room using the same type of CO_2_ analyzers as in the patient rooms (PP Systems SBA-5) connected to Onset HOBO U12 data loggers. The fraction of outdoor air delivered to each floor was calculated using a combination of CO_2_ data from all three air streams from each AHU [22]. Uncertainty in each interval estimate of outdoor air fraction was estimated by adding the instrument uncertainty of the CO_2_ sensors in each air stream in quadrature [22].

Air temperature was also measured in the outdoor, return, and supply air streams using an Onset TMC20-HD temperature sensor connected to an Onset HOBO U12 data logger (±0.25°C from 0°C to 50°C). Relative humidity was measured in both return air streams with an Onset HOBO U12 data logger. Water vapor pressure was measured directly in the two supply air streams and in one outdoor air stream (AHU 6) using the SBA-5 monitors and compared to the saturation vapor pressure at each measured temperature to calculate relative humidity in these locations. These measurements were also synchronized with the patient room and nurse station measurements and logged at 5-minute intervals. The temperature and humidity measurements from each air stream also served to validate estimates of outdoor air fractions with data from the hospital facilities department, who occasionally provided information on air handling unit damper positions over select 24-hour periods. These data were used to calibrate outdoor air fraction estimates using a procedure described in the Supporting Information (SI).

Finally, only spot measurements of the airflow rates in individual patient rooms were made prior to the hospital opening due to the level of invasiveness required for these measurements. Both recirculated airflow rates and exhaust airflow rates were measured using a pressure-matching technique combined with a calibrated fan (Energy Conservatory Duct Blaster). Similar measurements were also performed at the two slot supply diffusers; although we have much more confidence in the return and exhaust flow measurements because of the simpler geometries involved. Additionally, several CO_2_ sensors were deployed during a short field campaign in February 2013 (prior to the hospital opening) to assess mixing characteristics in one of the patient rooms; results confirmed that the rooms were reasonably well-mixed due in large part to very high airflow rates relative to indoor volumes. The flow measurements are described in more detail in **Table A in S1 File**, along with methods for calibration and co-location of all relevant sensors (**S1 Fig.**).

### Analysis

Upon initial sensor deployment, data were collected on a weekly basis. All collected built environment data was managed and analyzed using the data analysis and statistical software package Stata Version 13. Data recorded within 15 minutes of the weekly data collection period for each sensor and logger combination were excluded to account for the time it takes to disconnect and reconnect data loggers. The resulting dataset was then explored to address the research questions described herein.

## Results

### Built Environment Data Summary

The patient room and nurse station measurements produced approximately ∼7.26 x10^6^ data points over the course of the project (**Table 1**). The hospital was relatively tightly controlled for thermal comfort, with the majority (i.e., the interquartile range, IQR) of patient room and nurse station air temperatures falling within a 2.1°C range. Room air relative humidity and humidity ratio showed greater variation than temperature, but were still relatively tightly controlled; the IQR for relative humidity and humidity ratio spanned ∼10% and ∼1.6 g_w_/kg_da_, respectively (the relative standard deviation of each of these parameters was also larger than that of air temperature). The wide range of illuminance values (i.e., an IQR spanning ∼165 lx) accounts for minimal light during nighttime hours and much higher levels during daytime hours with high solar insolation penetrating the glazing. Values for both room air CO_2_ concentrations and IR beam-break counts varied widely during the 5-minute measurement intervals, with the IQR spanning a range of 53 ppm and 1 count, respectively (there were no beam-breaks recorded in the doorways during more than half of the logged 5-minute intervals). Finally, differential pressure measurements revealed that the rooms were indeed neutral pressure environments, with a mean near 0 Pa and an IQR spanning only 0.9 Pa (these values are all within or near propagated instrument uncertainty).

**Table 1:** Summary statistics for all parameters measured within all 10 patient rooms and two nurse stations at 5-minute intervals.

Parameter	N	Mean	s.d.	1%	10%	25%	50%	75%	90%	99%
Temperature (°C)	1.20×10^6^	23.5	1.4	19.9	21.7	22.6	23.7	24.7	25.2	25.9
Relative humidity (%)	1.20×10^6^	34.8	6.8	14.3	27.2	30.9	35.4	39.5	42.5	48.5
Humidity ratio (g_w_/kg_da_)	1.20×10^6^	6.3	1.2	2.4	5.0	5.6	6.4	7.2	7.4	7.7
Illuminance (lx)	1.20×10^6^	173	448	3.9	11.8	11.8	59.1	177	437	1478
IR beam-break (counts/5 min)	9.46×10^5^	0.74	1.68	0	0	0	0	1	3	8
CO_2_ (ppm)	8.13×10^5^	416	40	343	369	388	413	441	468	524
Differential pressure (Pa)	7.06×10^5^	0.1	0.6	−1.1	−0.8	−0.3	0.1	0.6	0.8	1.1
Total	7.26×10^6^									

The HVAC system measurements produced an additional ∼1.64 x10^6^ data points made in the mechanical room, including CO_2_ concentrations, air temperature, relative humidity, and humidity ratio in the three airstreams (**Table 2**). Outdoor air temperatures ranged from −14.6°C to +31.0°C across the year. Return air and supply air temperatures were much more consistent with median values (and IQR ranges) of 23.1°C (0.4°C) and 12.1°C (0.7°C), respectively. Outdoor relative humidity and humidity ratio varied widely in the Chicago climate, while return and supply air relative humidity and humidity ratio reflected a combination of influences from outdoor air fractions and cooling operation. Outdoor air and supply air CO_2_ concentrations were consistently lower than return air CO_2_ concentrations, suggesting that the HVAC systems often operated closer to 100% outdoor air than 100% return air (this was also verified by the schedule of operation from the facilities department). Supply air CO_2_ concentrations were often lower than outdoor air CO_2_ concentrations; however this should not be the case and this anomaly is likely explained by instrument uncertainty, as the differences between supply air and outdoor air CO_2_ concentrations were almost always within propagated instrument uncertainty. Therefore, outdoor air fractions were difficult to estimate accurately given the very small concentration differences observed. However, we explore an alternate estimation procedure for outdoor air fractions and also compare these estimates to damper information from the building facilities department in the SI (**S2 and S3 Figs.**).

**Table 2:** Summary statistics for all parameters measured at 5-minute intervals in mechanical rooms.

Parameter	N	Mean	s.d.	1%	10%	25%	50%	75%	90%	99%
CO_2_ concentration (ppm)										
Outdoor air**	8.12×10^4^	379	22	330	351	367	378	393	406	437
Return air*	1.62×10^5^	434	22	389	408	419	432	448	464	492
Supply air*	1.53×10^5^	365	21	325	339	350	363	379	492	421
Temperature (°C)										
Outdoor air	1.81×10^5^	11.2	10.9	−14.6	−3.1	2.6	11.8	20.5	24.8	31.0
Return air *	1.07×10^5^	23.1	0.3	22.5	22.8	22.9	23.1	23.3	23.5	23.9
Supply air *	1.89×10^5^	12.3	0.8	11.0	11.8	11.9	12.1	12.6	13.0	15.4
Relative humidity (%)										
Outdoor air **	9.05×10^4^	76.4	58.1	25.7	35.6	44.0	58.3	91.8	>100	>100
Return air *	1.07×10^5^	34.6	6.6	19.4	28.0	29.7	31.5	41.7	43.4	44.7
Supply air *	1.85×10^5^	69.5	14.9	33.0	51.4	56.8	69.5	83.2	86.8	93.1
Humidity ratio (g_w_/kg_da_)										
Outdoor air **	9.05×10^4^	6.2	2.4	2.9	3.6	4.1	5.6	8.0	9.5	12.3
Return air *	1.07×10^5^	6.1	1.2	3.4	4.9	5.2	5.5	7.4	7.6	7.8
Supply air *	1.85×10^5^	6.1	1.3	2.9	4.6	5.0	6.2	7.2	7.6	8.3
Total	1.64×10^6^									

*Measured in both air handling unit 6 (AHU 6) and air handling unit 11 (AHU 11).

**Measured in AHU 6 only.

### Indoor Environmental Conditions: Temporal Variations and Statistical Distributions

**Fig 2:**
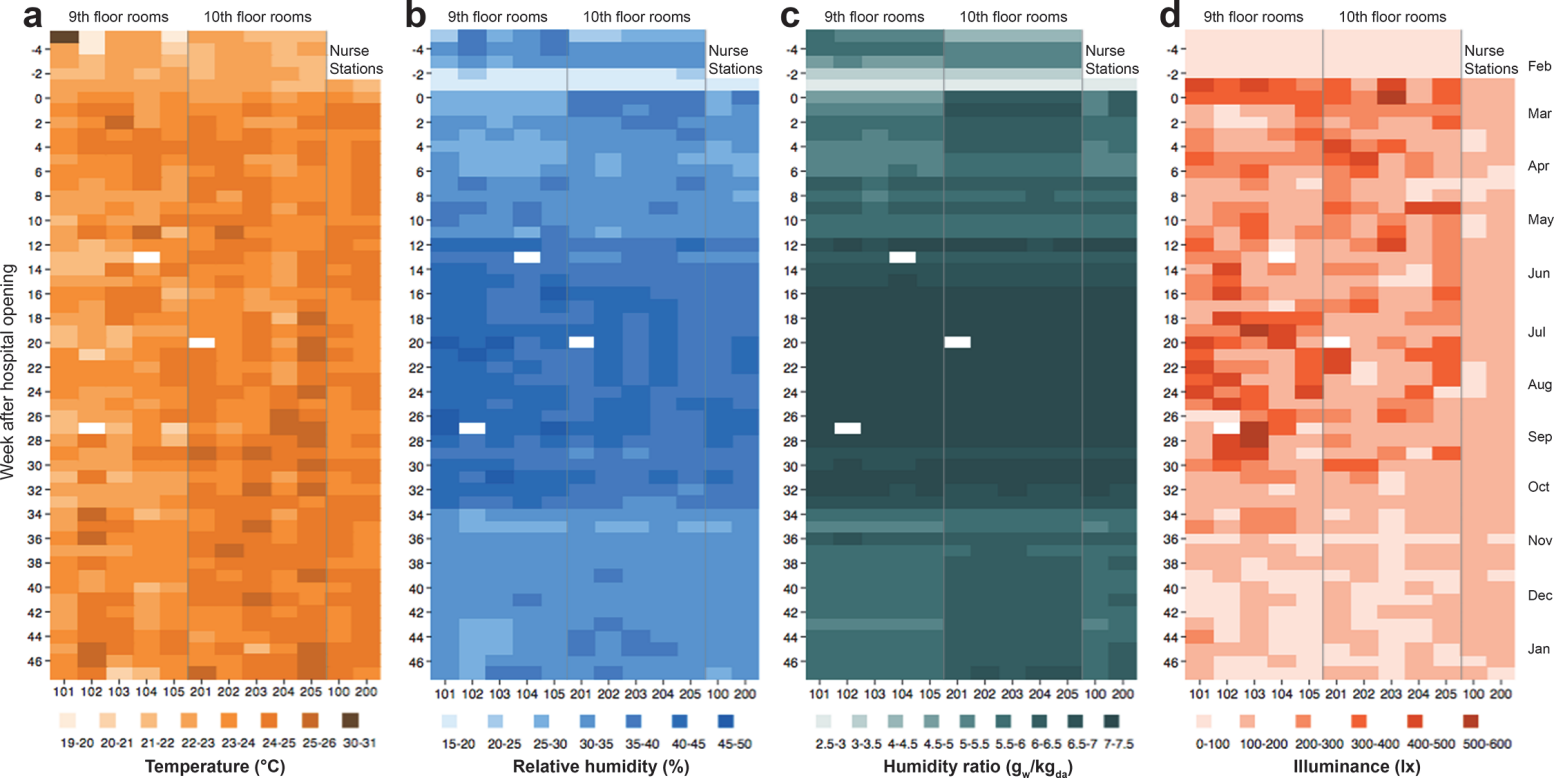
Weekly averages of environmental conditions in the patient rooms and nurse stations measured over the duration of the project: (a) temperature, (b) relative humidity, (c) humidity ratio, and (d) illuminance. Rooms 101–105 are on the 9^th^ floor; rooms 201–205 are on the 10^th^ floor. Room 100 and 200 are the nurse station locations on the 9^th^ and 10^th^ floor, respectively. Weeks are counted from the week of hospital opening (i.e., week 0). White areas represent missing values. Values along the x-axes correspond to room identification numbers. Examples time series data at 5-min intervals for one day are shown in S5 Fig.

**Fig 3:**
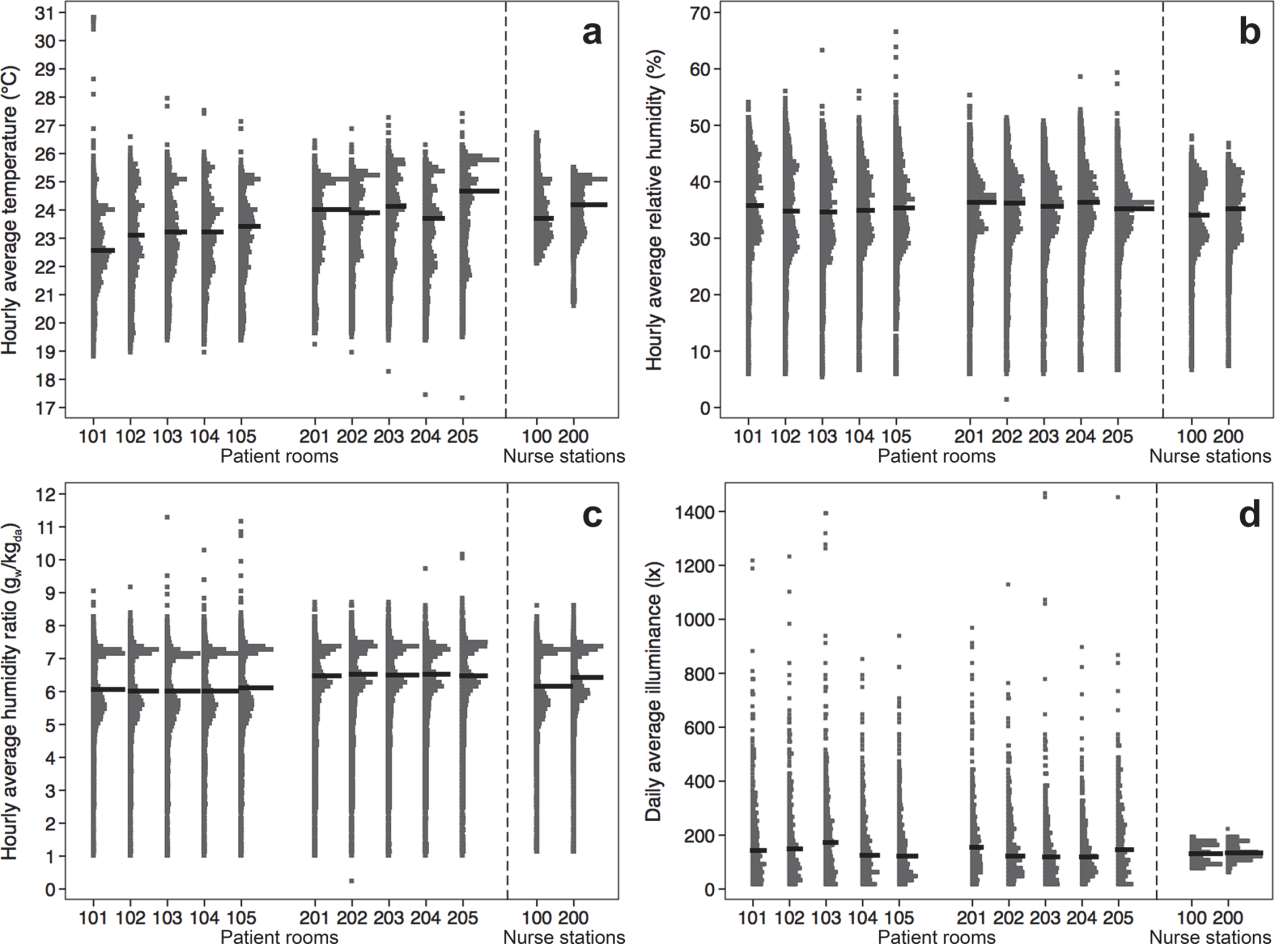
Distribution dot plots for four measured environmental conditions in the hospital: (a) hourly average air temperature, (b) hourly average relative humidity, (c) hourly average humidity ratio, and (d) daily average illuminance levels. Rooms 101–105 are on the 9^th^ floor; rooms 201–205 are on the 10^th^ floor. Room 100 and 200 are the nurse station locations on the 9^th^ and 10^th^ floor, respectively. The horizontal black bar represents the median hourly values (only daily averages are provided in part (d) for better graphical clarity).

Weekly average air temperatures ranged from 19.6°C to 30.1°C across all patient rooms and from 21.0°C to 24.9°C at the two nurse stations (**Fig. 2A**). Hourly average air temperatures across all patient rooms typically varied between approximately 19°C and 26°C (a range of about 7°C), with median hourly temperatures ranging between 22°C and 25°C (**Fig. 3A**). The observed variation likely stems from patient comfort preferences, as each patient had the ability to control their room air temperature. Nurse stations appeared to be more centrally controlled, with hourly average air temperatures spanning a range of only about 4°C. Across all rooms, there was no correlation between hourly average indoor air temperatures and outdoor air temperatures (**S6A Fig.**), suggesting that outdoor temperatures had no influence on indoor temperatures. Slight differences in indoor air temperatures between the lower floor and upper floor were apparent in both patient rooms and nurse stations, with median temperatures about 1°C higher on the upper floor, suggesting an influence of the HVAC systems or building design. One point of distinction occurred in Room 101, in that several hourly data points were greater than 30°C (well beyond the zone of thermal comfort [23]); although these temperatures were observed prior to the hospital opening and can be attributed to systems testing. Average daily air temperatures were also consistently higher after the hospital opened (Feb 24, 2013—Jan 15, 2014), with temperatures approximately 1.6°C lower before patients and staff were introduced (Jan 22—Feb 23, 2013), on average (p < 0.05 for all 12 location comparisons using a Mann-Whitney two-sample test). Air temperature distributions were either multi-modal or lacked a clear distribution shape in most patient rooms and nurse stations.

Weekly average relative humidity values ranged from ∼15% to 48% across all patient rooms and nurse stations (**Fig. 2B**). Hourly average relative humidity values (**Fig. 3B**) in both patient rooms and nurse stations demonstrated greater variation, from 10% to 60%; although lower values (<25%) occurred quite infrequently. The majority (between 88% and 95%, depending on sample location) of hourly average relative humidity measurements were between 25% and 50%, with median relative humidity values around 35% and values fairly consistent across all rooms and nurse stations. It is important to note the similarities in variation within patient rooms and nurse stations, indicating the strong effect from the hospital HVAC systems and little effect from individual microenvironments. Hourly relative humidity distributions typically consisted of two primary modes in most locations, albeit with some scatter. Daily averages of relative humidity were also significantly greater after the hospital opened (mean of approximately 37% compared to 25%; p < 0.05 for all 12 location comparisons using a Mann-Whitney two-sample test), although the differences were largely driven by seasonal variations. For example, there were only minor, non-significant differences in daily mean relative humidity values in the patient rooms before or after opening if data were restricted only to days in January of both 2013 and 2014 (the only shared month).

The humidity ratio at each interval was calculated using concurrent temperature and relative humidity data from patient rooms and nurse stations [24]. Weekly average humidity ratio values ranged from about 2.5 g_w_/kg_da_ to 7.5 g_w_/kg_da_ across all patient rooms and nurse stations (**Fig. 2C**). Variations in both weekly and hourly average humidity ratios within rooms and nurse stations appeared consistent and similar across all measurement locations, with the majority of measurements falling between 5 g_w_/kg_da_ and 8 g_w_/kg_da_ (**Fig. 3C**). However, there was noticeably less variation in humidity ratios (and to a lesser extent relative humidity) from weeks 16–28 (which occurred in the summer months of Jun—Sep 2013). In fact, there was no correlation between hourly average indoor humidity ratios across all rooms and nurse stations and outdoor humidity ratios between ∼8 and 14 g_w_/kg_da_, while there was a strong linear correlation between indoor and outdoor humidity ratios between 2 and 8 g_w_/kg_da_ (**S6B Fig.**). There was also a clear difference between floors, with indoor humidity ratios on the upper floor consistently greater than the lower floor (although the median difference in hourly averages was less than 0.5 g_w_/kg_da_). Hourly humidity ratio distributions consisted of two very clear modes in all locations, with peaks around 5.6 and 7.1 g_w_/kg_da_ on the lower floor and around 6.1 and 7.2 g_w_/kg_da_ on the upper floor. These data suggest that the influence of the HVAC system on indoor humidity ratio considerably overwhelmed influences from individual patient activities or thermostat control.

Weekly average illuminance levels ranged from approximately 10 lx to 590 lx across all patient rooms and from approximately 75 lx to 190 lx at the nurse stations (**Fig. 2D**). Distributions of illuminance levels in the patient rooms and nurse stations were summarized using daily averages instead of hourly averages (hourly variations were quite extreme due to large differences between daytime and nighttime). Daily average illuminance levels ranged from 0 to 1400 lx across all sampling locations, but the majority of daily averages were less than 200 lx (**Fig. 3D**). Median values were similar across all locations as well, although all patient rooms (which were all west-facing rooms with large windows) had several days with much higher illuminance. Daily mean illuminance levels at the nurse stations (which received only artificial light) clustered between about 100 and 200 lx, suggesting that the large values observed in patient rooms potentially resulted from periods of high solar radiation. Indeed, illuminance levels in patient rooms increased most during the afternoon hours, with increasing solar radiation during this time of day on the west-facing facades (although occupant shade control led to some variations in magnitude and timing). Additionally, nurse station lights were dimmed rather than shut off during evening hours, so nurse station illuminance levels were never as low as observed for patient rooms.

### Surrogates for Daily Human Occupancy/Activity: Temporal Variations and Statistical Distributions

**Fig 4:**
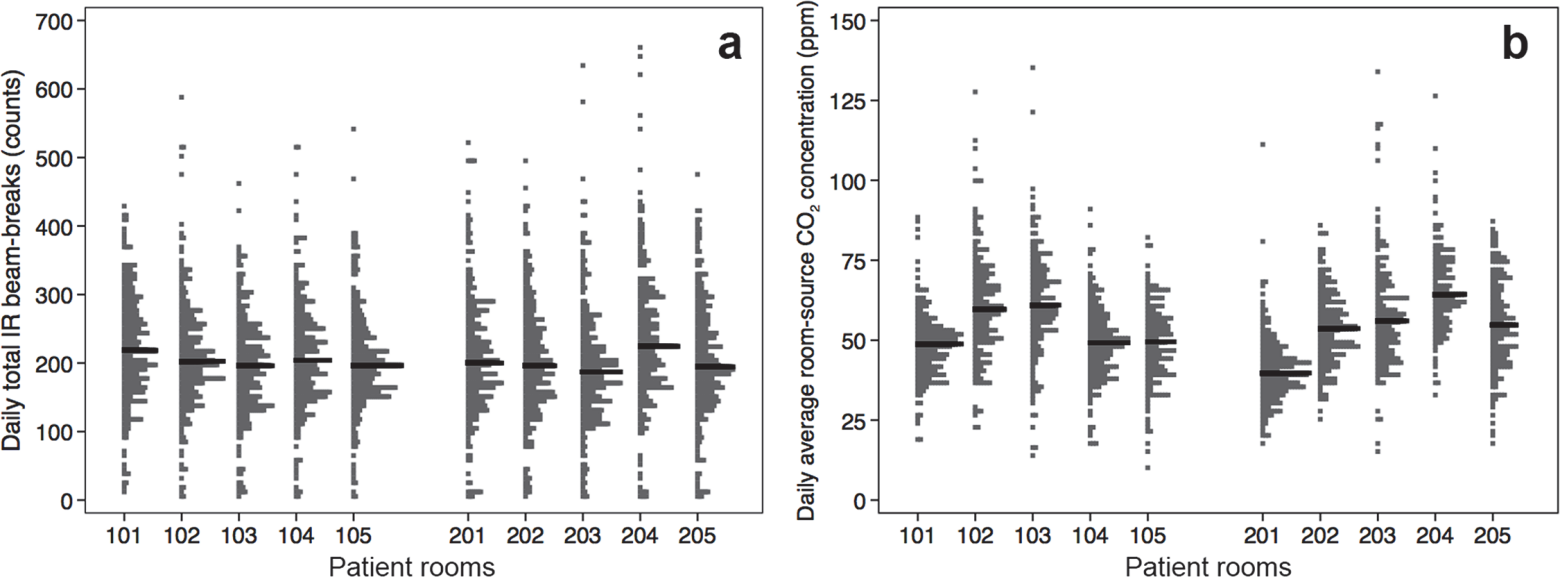
Surrogates for daily human occupancy and activity: (a) daily total IR bream-break counts and (b) daily average room-source CO_2_ concentrations. Rooms 101–105 are on the 9^th^ floor; rooms 201–205 are on the 10^th^ floor. The horizontal black bar represents the median daily values

Daily total beam-break counts demonstrated similar variance across all patient rooms (**Fig. 4A**), with median daily total counts between 175 and 225 for all rooms. Assuming that half of the beam-breaks over the course of a day represent entrances and half represent exits, the median number of combined entrances and exists was typically between 90 and 110 in the patient rooms (this method does not count individuals but does provide a sense of overall activity into and out of the room). Over all rooms, few days had less than 100 beam-breaks (i.e., 50 pairs of combined entrances and exits), indicating that all patient rooms typically averaged at least 4 beam-breaks per hour (i.e., 2 pairs of combined entrances and exits), on average.

Measurements of both occupancy/activity surrogates in room 204 appeared to be slightly higher than those in all other rooms, with medians noticeably higher than all other rooms and several days with total beam-break counts over 500 (i.e., 250 pairs of combined entrances and exits). This was largely driven by a small number of high occupancy periods during a few days in July and October 2013, although the cause of this elevated occupancy/activity cannot be explained by our data. Additionally, the introduction of patients and staff clearly influenced the beam-break occupancy surrogates as expected. There were typically 2.5–3 times as many daily total beam-breaks in each room after the hospital opened in February 2013 (mean across all rooms of ∼211 after occupation and ∼74 before occupation; p < 0.0001 for all 10 comparisons using a Mann-Whitney two-sample test).

Median values of daily average room-source CO_2_ concentrations were relatively similar among rooms, ranging between ∼40 and ∼61 ppm (**Fig. 4B**). However, propagated instrument uncertainty was as high as 28 ppm for each sensor. This maximum value is determined by adding the CO_2_ sensor uncertainty reported by the manufacturer (20 ppm) and data logger uncertainty (2 mV ± 2.5% absolute reading) in quadrature (and assuming a higher range of raw CO_2_ values of approximately 700 ppm). Using that maximum value, the propagated uncertainty in *C*_*room-source*_ was estimated to be approximately 40 ppm by adding the compounded uncertainties for individual sensors used in pair-wise comparisons in quadrature. However, the use of the same CO_2_ sensors in each patient room throughout the project provides greater confidence in these data (through internal comparisons), which suggest that distributions of room-source CO_2_ concentrations were wider in some rooms and implies that occupancy patterns demonstrated greater variation in those rooms (e.g., Room 203 versus Room 204, immediately adjacent).

**Fig 5:**
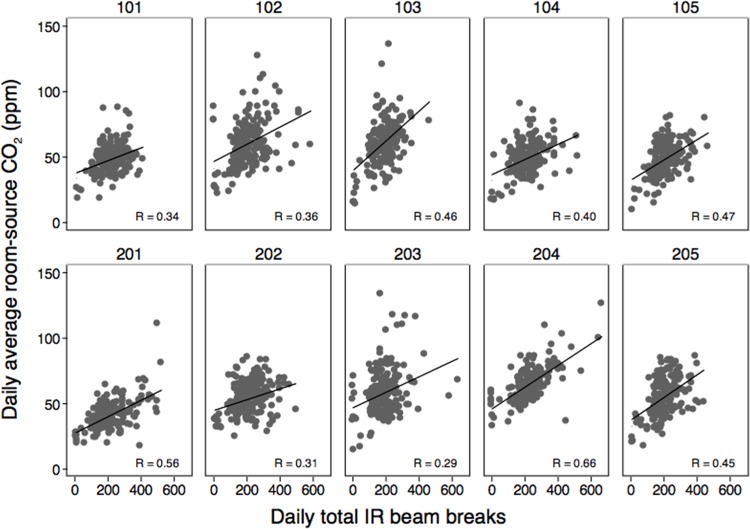
Estimated daily average room-source CO_2_ measurements and daily total IR beam-breaks in the patient rooms. Rooms 101–105 are on the 9^th^ floor; rooms 201–205 are on the 10^th^ floor. Room 100 and 200 are the nurse station locations on the 9^th^ and 10^th^ floor, respectively. Pearson correlation coefficients are shown. All ten correlations are statistically significant at p < 0.001. Headings above each graph correspond to patient room numbers.

We also explored correlations between average daily occupancy based on room-source CO_2_ concentrations and daily total IR beam-break counts (**Fig. 5**). The two surrogate measures in each patient room were reasonably well correlated with statistically significant relationships in all comparisons and correlation coefficients ranging from 0.29 to 0.66. These data suggest that both sensors may be able to serve as reasonable indicators of human occupancy in the patient rooms. In theory, the room-source CO_2_ measurements could provide accurate estimates of occupancy using a mass balance approach; however, this method suffers from large uncertainty and a number of instrumentation reliability issues (e.g., the number of data points for comparison was limited primarily by a smaller number of valid CO_2_ measurements available due to a combination of instrumentation issues). Conversely, the IR beam-break sensors proved to be more robust and seldom had missing data; however, daily beam-break totals are not a direct measure of average occupancy, and are a better surrogate for overall occupant activity.

### Spatial Variations Between Locations: Patient Rooms and Nurse Stations

#### Environmental Conditions

The majority of comparisons of daily mean air temperature between all locations have an *R* < 0.2, with the exception of a few room combinations (**S7 Fig. and Table B in S1 File**). However, there were no significant correlations if comparisons are limited only to measurements made after the hospital was occupied, suggesting that any significant correlations were driven by those that occurred prior to the hospital opening when there were no occupants controlling temperature for comfort. Overall, ∼28.7% of all measured differences in daily average air temperatures were found to be within the range of propagated instrument uncertainty (0.57°C, estimated by adding the manufacturer-reported uncertainty of each temperature sensor (0.4°C) in quadrature), with 18.1% to 42.9% of measurement differences falling within this range of uncertainty for individual room comparisons (**Table D in S1 File**). Additionally, we have good confidence that the actual uncertainty in the temperature measurements is lower according to our co-location calibration experiments. Therefore, the weak correlation observed is believed to largely stem from true differences in daily mean temperatures and not sensor uncertainty.

In contrast with air temperatures, strong correlations in daily mean relative humidity values were observed in patient rooms and nurse stations on both floors, with correlation coefficients typically between 0.7 and 0.8 (**S8 Fig. and Table B in S1 File**). While all room comparisons generated significant correlations for relative humidity values, stronger correlations existed between rooms on the same floor (*R* > 0.8). These strong correlations between sample locations indicate that the largest influence on room relative humidity values was the common HVAC system, with little effect from environmental conditions, occupancy, or occupant activity. Overall, ∼66.8% of all measured relative humidity differences were found to be within the range of propagated instrument uncertainty (i.e., 3.5%, estimated by adding the manufacturer-reported uncertainty of the relative humidity sensors [2.5%] in quadrature), with 48.8% to 84.5% of measurement differences falling within this range of uncertainty for individual room comparisons (**Table D in S1 File**). About 75% of the daily average measurements fell within this range of uncertainty within patient rooms and nurse stations on the same floor. The large number of comparisons being within the range of instrument uncertainty supports the strong correlations.

Daily average humidity ratios showed even stronger correlations between rooms and nurse stations (**S9 Fig. and Table B in S1 File**). As with relative humidity, room humidity ratio comparisons were again all significant, and the highest correlations also occurred between rooms on individual floors. The nurse station and rooms on the same floors all had correlation coefficients of at least 0.99. Correlations were slightly weaker between floors. About 63.4% of all measured humidity ratio differences were found to be within the range of propagated instrument uncertainty (approximately 0.14 g_w_/kg_da_, estimated by calculating the impact of uncertainty from both the individual temperature and relative humidity sensors on humidity ratio, ∼0.1 g_w_/kg_da_, and adding that value in quadrature), with 24.4% to 98.1% of measured differences falling within this range of uncertainty for individual location comparisons (**Table D in S1 File**). Within patient rooms and nurse stations on the same floor, about 85% of the daily average measurements fell within the range of uncertainty. The large number of comparisons with differences within the range of instrument uncertainty again supports the strong correlations between humidity ratio measurements. These data further suggest that absolute humidity in the hospital was tightly controlled by the HVAC systems and that the HVAC systems on both floors operated in a similar fashion.

Daily average illuminance levels between rooms on the same floor appeared to show moderate correlations, particularly at lower illuminance levels (**S10 Fig. and Table B in S1 File**). No correlation was apparent between nurse stations and rooms on the same floor, with nurse station illuminance levels remaining relatively low compared to rooms, as expected due to the absence of immediate external windows in nursing stations. Correlation coefficients across all patient rooms and nurse stations reveal a wide variation both within and between floors. Correlation coefficients were as low as 0.18 between patient rooms and as high as 0.55 between floors; although nearly all correlations between patient rooms were statistically significant. Nurse stations on both floors were not significantly correlated with either other patient rooms or the other nurse station. Uncertainty in illuminance measurements was not explored because the manufacturer did not report sensor accuracy.

#### Patient Room Occupancy/Activity

Correlations between daily total IR beam-break counts among individual rooms were extremely weak and mostly insignificant (**S11 Fig. and Table C in S1 File**). However, correlations were slightly stronger for rooms on the same floor as opposed to rooms between different floors. Between floors, correlation coefficients ranged from as low as 0.02 to as high as 0.34. Only 9 out of 45 room comparisons (20%) revealed significant correlations. Uncertainty in IR beam-break measurements was not explored because the nature of the measurement did not allow for direct comparisons. Overall, very weak correlations were observed between daily total IR beam-breaks in individual rooms, suggesting that the patient rooms were independently highly variable in terms of human occupancy and activity.

Room-source CO_2_ concentrations revealed weak correlations between patient rooms (**S12 Fig. and Table C in S1 File**). Only 8 out of 45 room comparisons (18%) showed statistically significant, albeit weak, correlations. Only three of these significant correlations came from the same patient rooms that had significant correlations in daily total IR beam-breaks, suggesting that the two methods cannot be used interchangeably to assess occupancy. An average of 94.3% of all measured differences in daily average room-source CO_2_ concentrations between rooms was found to be within the range of propagated uncertainty (estimated as 40 ppm, as previously discussed), with 86.7% to 100% of measured differences falling within this range of uncertainty for individual room comparisons (**Table D in S1 File**). That the vast majority of values were within instrument uncertainty limits confidence in our ability to distinguish differences in room-source CO_2_ concentrations (and associated occupancy estimates) between patient rooms.

### Spatial Variations Between Floors

**Fig 6:**
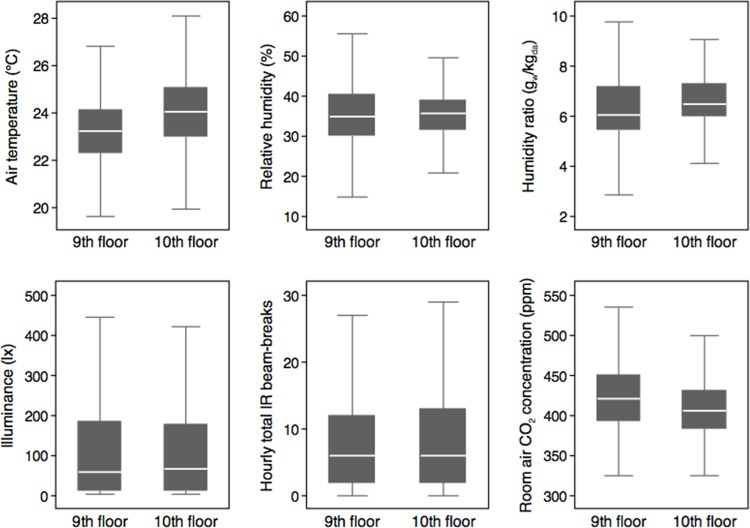
Variations between floors for hourly average air temperature, relative humidity, humidity ratio, illuminance, and room CO_2_ concentration, as well as hourly total IR beam-breaks. Box plots display median values in the center, bounded by the interquartile range in gray and extreme values at top and bottom. Figures exclude outlier values for clarity.

The range of daily average air temperatures was slightly higher on the upper floor compared to the lower floor, with both higher median (∼24.1°C vs. ∼23.2°C, respectively) and maximum temperatures (∼28°C vs. ∼27°C, respectively; **Fig. 6**). Conversely, humidity ratios had a larger range on the lower floor and slightly lower median values compared to the upper floor. The difference is intuitive given the effect from the HVAC systems and the fact that the two floors are served by different air handling units that operated at different outdoor air fractions during some outdoor climate conditions. The lower floor had both a lower median outdoor air fraction and a wider range of outdoor air fraction values (**S4 Fig.**), which is consistent with information obtained from the building’s facilities manager. Daily illuminance levels between floors was very similar, which is reasonable given that all rooms were west-facing and have similar elevations and fenestration ratios. Distributions of hourly IR beam-break counts and CO_2_ concentrations were very similar between floors, indicating that aggregate occupancy and activity between floors were very similar (although concurrent between-room as well as within-room differences have already been shown to be quite large).

### Temporal Variations (Daytime vs. Nighttime)

**Fig 7:**
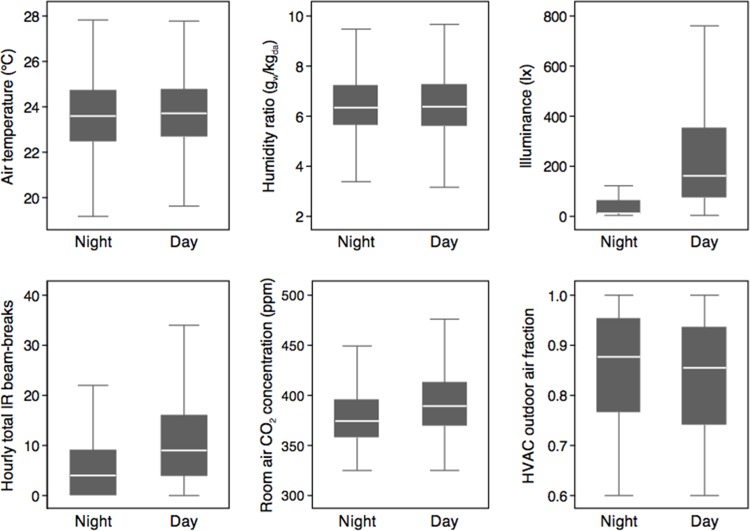
Variations between nighttime and daytime periods for hourly average air temperature, humidity ratio, illuminance, room CO_2_ concentration, and outdoor air fraction, as well as hourly total IR beam-breaks. Box plots display median values in the center, bounded by the interquartile range in gray and extreme values at top and bottom. Figures exclude outlier values for clarity.

Hourly average air temperature, humidity ratio, relative humidity (not shown but very similar to humidity ratio), and outdoor air fractions revealed no differences between nighttime and daytime periods (with night defined as 7:00pm to 6:59am and day defined as 7:00am to 6:59pm; **Fig. 7**). However, as expected, illuminance levels were much higher in all locations during daytime periods (median values of nearly 200 lx during daytime and under 25 lx during nighttime periods) across all locations. Also quite intuitively, both hourly total IR beam-break counts and hourly average CO_2_ concentrations in the patient rooms were moderately higher during daytime periods (median of approximately 9 vs. 4 hourly beam-breaks and ∼390 ppm vs. ∼375 ppm for CO_2_ concentrations during daytime vs. nighttime periods, respectively).

## Discussion

The Hospital Microbiome Project (http://www.hospitalmicrobiome.com) is a multidisciplinary study of the hospital built environment. While efforts are still underway to describe the ecology of microbial communities that colonized and spread throughout the hospital, the analysis of the built environment data herein can provide insight into the associated dynamics of this new building. The built environment data collection campaign generated approximately 8×10^6^ data points covering a range of building environmental and operational parameters that may have influenced occupant comfort and health, indoor environmental quality, and the growth, survival, and succession of microbes within the building. Within-room, between-room, between-floor, and temporal variations in individual parameters revealed interesting, sometimes intuitive, patterns. For example, patient room and nurse station air temperatures varied more than expected for such a typically tightly controlled environment (although the variation was still over a relatively tight range, consistent with that of modern commercial and institutional buildings). Effects from occupant temperature control clearly contributed to the range and variation within patient rooms and thus the low correlations between rooms and nurse stations. Relative humidity and humidity ratios were both strongly correlated between patient rooms, indicating a strong effect from the HVAC system and very little effect from occupants and activities, particularly for humidity ratio, which is not dependent on temperature. The time-series data indicated that the indoor humidity ratio was tightly controlled during summer and, to a lesser extent, during winter months when the weather was most extreme in Chicago. Between those seasons, humidity ratios varied more widely, indicating a lack of central control during transition months and swing seasons. However, the HVAC system appeared to be designed and operated to limit indoor humidity ratios to less than ∼9 g_w_/kg_da_, regardless of how high outdoor humidity ratios reached (**S6 Fig.**). Illuminance levels showed moderately strong correlations between the perimeter patient rooms and weak correlations to the interior nurse stations, which is likely attributed to similar solar exposure on the west-facing facades among all patient rooms. Differences between patient rooms likely also stemmed from occupant control over both window shades and artificial lighting.

It is important to note that although similar distributions and/or median values of hourly or daily measurements of certain indoor environmental parameters were observed in all or most locations over the course of the project, certain parameters exhibited more variation between locations temporally (e.g., on a weekly timeframe). For example, the distributions and median values of hourly average relative humidity appear similar across all locations, whereas the weekly average relative humidity values exhibit more variation between locations.

The combination of “room-source” CO_2_ concentrations and IR beam-break data appeared to serve as useful surrogates for occupancy and activity. Daily averages of patient room-source CO_2_ concentrations and daily totals of IR beam-break counts were reasonably well correlated (more so in some rooms than others), suggesting that we were able to provide some helpful metric of both occupancy and activity. With a median of approximately 200 doorway beam-breaks per day (i.e., 100 pairs of combined entrances and exits per day), the patient rooms appear to be relatively lightly occupied compared to other types of environments, such as retail buildings, but tend to have fewer vacant periods than many offices of similar sizes [25,26].

### Implications for Human Comfort

Overall, many of the indoor environmental conditions were fairly consistent with other long-term or large-sample studies. For example, measured air temperatures, relative humidity, and humidity ratios were generally in the range of those measured in a large sample of nearly 100 U.S. office buildings [27], but temperatures were grouped more tightly than is typically seen in U.S. residences [28]. The majority of measured temperature and relative humidity values were in the range of acceptable thermal comfort [23]. Measured illuminance levels in the patient rooms were quite similar to the range observed in a large study of Hong Kong apartments and are also within the range considered “acceptable” [29].

### Implications for Indoor Environmental Quality

Total airflow rates and outdoor air ventilation rates in the patient rooms appeared to exceed minimum requirements for treatment rooms and critical care rooms in ASHRAE Standard 170 [30], which defines ventilation system design requirements that provide environmental control for comfort, asepsis, and odor in healthcare facilities. Design total supply airflow rates ranged from 660–765 m^3^/hr, providing ∼7–8 air changes per hour (ACH) of HEPA-filtered supply air in each room (Standard 170 requires 6 ACH [30]). Design outdoor air fractions ranged from ∼61–100% on the lower floor and 75–100% on the upper floor, providing design outdoor air ventilation rates ranging from ∼4 to ∼8 ACH throughout the project duration (Standard 170 requires 2 ACH [30]).

### Implications for the Survival and Succession of Microbial Communities

Environmental conditions such as temperature, relative humidity, and humidity ratio have been shown to influence both surface-bound and airborne microbes in varying ways, including varying growth or survival responses by viruses, bacteria, and fungi (including infectious agents) under different conditions [12,31–46]. One recent study in U.S. hospitals found that the summer season (which may be associated with different HVAC operational patterns, similar to those observed herein) and higher mean monthly outdoor temperatures were both associated with increased frequency of bacterial bloodstream infections among hospitalized patients [47].

The range of air temperatures measured in this project (which were also shown to correlate well to many surface temperatures early in this project) was typically only 7°C (from 19°C to 26°C) across all patient rooms, and even smaller at the nurse stations. It is not immediately clear how these small-magnitude differences may have influenced microbial growth/survival or infectious disease transmission, although this is the focus of an ongoing investigation. Hourly average relative humidity values ranged as much as 50% absolutely, with the majority of measurements between 25% and 50% across all patient rooms and nurse stations. Variation in humidity ratios was similar, with the majority of measurements occurring between 5 g_w_/kg_da_ and 8 g_w_/kg_da_. This relatively large range of relative humidity and humidity ratio values is likely to have influenced both viral and bacterial survival in the hospital, given findings from a number of controlled studies in the literature [32,36–46]. However, because relative humidity and humidity ratio values correlated strongly between rooms, room-to-room variations in pathogen survival due to differences in humidity were likely small. Between-room differences in illuminance levels (particularly in response to greater amounts of sunlight penetrating the patient rooms) may also have influenced microbial survival because sunlight (through UV exposure) is known to inhibit bacterial growth or have bactericidal powers, even through panes of glass [48] (although modern glazing units are likely to have different effects).

Perhaps the largest potential contributor to between-room variation in indoor environmental quality and microbial community structure are the human occupants [49–51]. Occupant comfort and environmental preference influences environmental conditions (e.g., temperature, relative humidity, humidity ratio, and illuminance levels), which can potentially affect the progression and survival of microbial communities (as described above). Furthermore, large differences in occupancy and activity between rooms (as well as large differences before and after the hospital opened) are likely to have influenced the abundance and composition of human-related microbial communities in the patient rooms and nurse stations [50–53]. However, the combination of beam-break and room-source CO_2_ data did not provide any information regarding occupant identification or periods of patient room turnover, which have been shown to be important for transferring bacterial communities (including antibiotic resistant strains) and increasing infection risk from one occupant to the next [54–56]. These data also did not provide any information on patient or visitor activity or mobility within the rooms, which is important for surface contamination and resuspension of settled floor dust [50].

### Implications for Other Built Environment Characterization Studies

We have demonstrated a systematic way to gather potentially valuable building environmental data with minimal costs and invasiveness, but at very high temporal and spatial resolution within the hospital. The use of off-the-shelf sensors to measure temperature, relative humidity, and illuminance levels with built-in data logging capabilities reduced the entailed cost and maintenance throughout the project. The IR beam-break people counters entailed lower costs and provided insight on human activity, but also gave a less accurate measure of occupancy, due to the non-directional rather than bi-directional functionality. CO_2_ sensors were useful in supplementing the people counters to measure occupancy, but had much higher costs and more downtime for maintenance due to additional components such as an absorber column and sampling lines. Additionally, their relatively high power requirements meant we were limited to sampling where electrical power was available. Finally, differential pressure sensors used were relatively low cost (although more than the temperature, relative humidity, and illuminance sensors), but also required an external data logging device, large battery packs, and external sampling lines.

It should be noted that the findings from this investigation are subject to a certain degree of error and uncertainty from factors such as sensor accuracy and uncertainty, measurement error (e.g., challenges when taking supply flow measurements), and others. Furthermore, the large number of data points taken and comparisons made can affect the results of statistical analyses (e.g., significance). However, the results from this investigation can be accepted with a reasonably high degree of accuracy and provide extensive information on building environmental and operational parameters and a robust characterization of a typical hospital environment.

Overall, using a suite of off-the-shelf sensors, we were able to measure multiple parameters continuously at 5-minute intervals over the span of approximately one year, providing millions of built environment data points for future comparisons. While this suite of measurements and methods provided a large robust dataset, additional development of lower cost sensors using common data collection platforms and remote uploading to the Internet should be prioritized in future investigations. Other possible improvements include using directional IR beam-break counters or other methods for assessing human occupancy, the installation of pressure taps to measure any changes in supply and recirculated airflow rates over time, measurements of equilibrium relative humidity on sampled surfaces (a surrogate for water activity), and more detailed measurements of human proximity near sample surfaces. The findings and recommendations in this study can be used to inform future investigations, and thus improve our understanding of the built environment.

## Supporting Information

S1 Fig Calibrated CO_2_ measurements in one unoccupied room on February 16, 2013, prior to hospital opening. CO_2_ measurements were made (1) in the center of the room, (2) near the west-facing window, (3) under a cabinet (where CO_2_ sensors were eventually installed for the long term measurements), and (4) on the countertop near the doorway.
(TIF)

S2 Fig Outdoor air (OA) fraction averaged over outdoor air temperatures for (a) air handling unit 6 (AHU 6) and (b) air handling unit 11 (AHU 11). The HVAC systems operated in economizer mode, varying OA fraction with outdoor temperature conditions.
(TIF)

S3 Fig Adjusted outdoor air (OA) fractions for air handling unit 6 (AHU 6) and air handling unit 11 (AHU 11) after applying calibration factors. The HVAC systems operated in economizer mode, varying OA fraction with outdoor temperature conditions.
(TIF)

S4 Fig Distribution of outdoor air (OA) fraction estimates across floors. OA fractions in the HVAC systems were typically lower on the 9^th^ floor than the 10^th^ floor.
(TIF)

S5 Fig Example 24-hour time-series built environment data (5-min intervals) in the hospital. Example time series of 5-minute data for temperature, relative humidity, humidity ratio, illuminance, doorway IR beam-breaks, room air CO_2_ concentration, pressure difference between room and hallway, and HVAC outdoor air (OA) fraction for one room on one floor.
(TIF)

S6 Fig Hourly average indoor environmental conditions for all patient rooms and nurse stations versus hourly average outdoor environmental conditions: (a) temperature and (b) humidity ratio. There was no correlation between indoor temperature and outdoor temperature. Indoor humidity ratio tracked outdoor humidity ratio until the outdoor humidity ratio reached ∼8 g_w_/kg_da_.
(TIF)

S7 Fig Variations in daily average air temperatures measured at each patient room and nurse station. Daily average air temperatures were weakly correlated between locations. The inset table shows pair-wise Pearson correlation coefficients for each location comparison.
(TIF)

S8 Fig Variations in daily average relative humidity (RH) measured at each patient room and nurse station. Daily average relative humidity measurements showed strong correlations between locations. The inset table shows pair-wise Pearson correlation coefficients for each location comparison.
(TIF)

S9 Fig Variations in daily average absolute humidity ratio measured at each patient room and nurse station. Daily average absolute humidity ratio measurements showed very strong correlations between locations. The inset table shows pair-wise Pearson correlation coefficients for each location comparison.
(TIF)

S10 Fig Variations in daily average illuminance measured at each patient room and nurse station. Daily average illuminance levels were moderately correlated between patient rooms, with no correlation between nurse stations. The inset table shows pair-wise Pearson correlation coefficients for each location comparison.
(TIF)

S11 Fig Variations in daily total doorway IR beam-breaks measured at each patient room doorway. Daily total doorway IR beam-breaks showed little correlation between patient rooms. The inset table shows pair-wise Pearson correlation coefficients for each location comparison.
(TIF)

S12 Fig Variations in daily average room-source CO_2_ concentrations measured at each patient room doorway. Daily average room-source CO_2_ concentrations showed little correlation between patient rooms. The inset table shows pair-wise Pearson correlation coefficients for each location comparison.
(TIF)

S1 File Supporting information includes data from instrument co-location, early measurements, QA/QC, example time-series data, and full correlation matrices. This file also contains Tables A-D. Table A, Flow schedule and baseline measurements. Table B, Pair-wise correlation matrices for daily mean air temperature, relative humidity, humidity ratio, and illuminance levels in the patient rooms and nurse stations. Table C, Pair-wise correlation matrices for daily total IR beam-breaks and daily average room-source CO_2_ (and occupancy) in the patient rooms. Table D, Fractions of measured differences in daily mean temperature, relative humidity, and humidity ratio between patient rooms and nurse stations, as well as room-air CO_2_ concentrations in the patient rooms, that were within the range of propagated uncertainty.
(DOC)
