# Supplementary figures and images for: Spatial and Temporal Variations in Indoor Environmental Conditions, Human Occupancy, and Operational Characteristics in a New Hospital Building
Source: PLoS One. 2015 Mar 2;10(3):e0118207. doi: 10.1371/journal.pone.0118207 (PMC4346405; doi:10.1371/journal.pone.0118207)

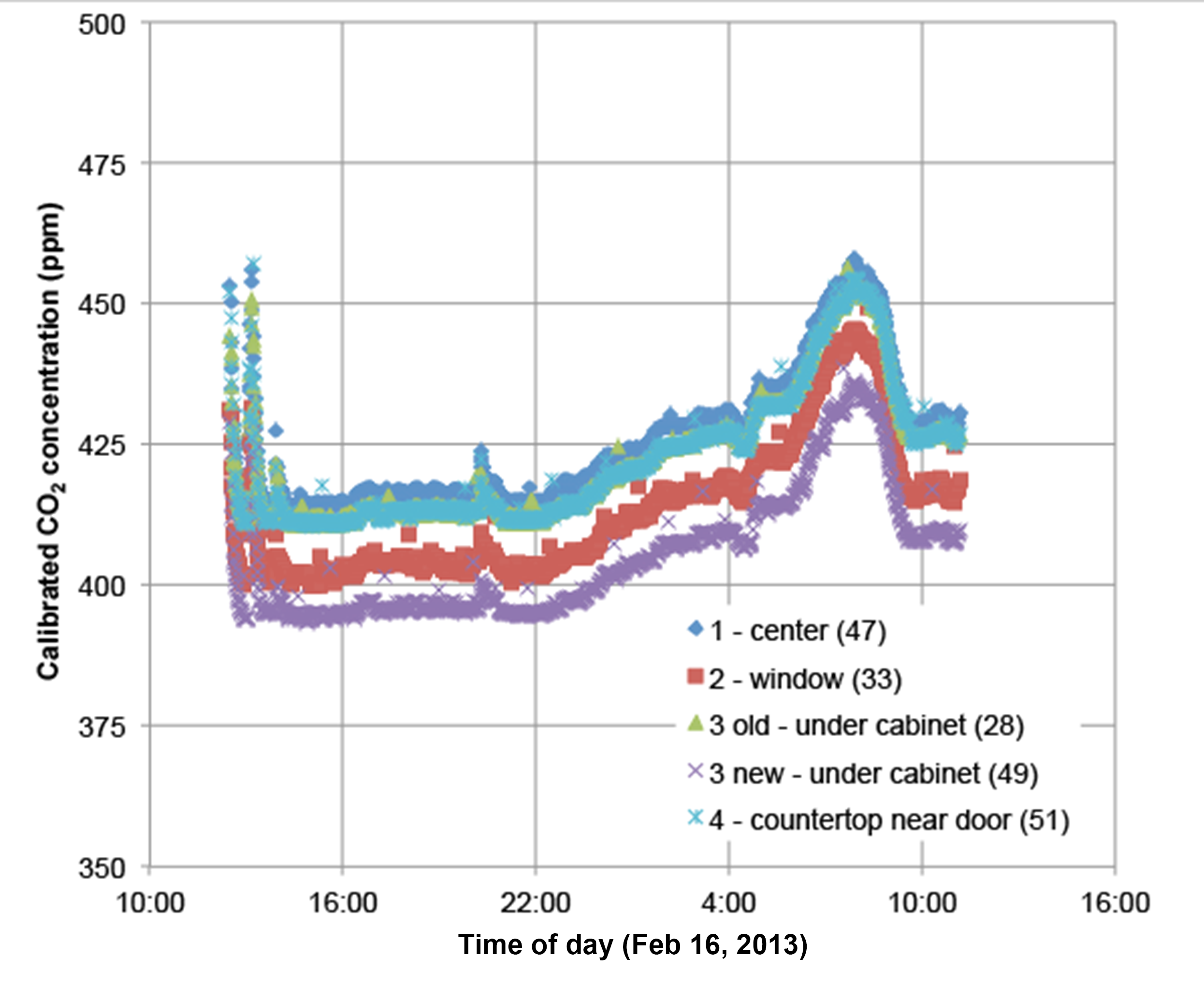

Supplement: S1 Fig — CO2 measurements were made (1) in the center of the room, (2) near the west-facing window, (3) under a cabinet (where CO2 sensors were eventually installed for the long term measurements), and (4) on the countertop near the doorway. (TIF) [file pone.0118207.s001.tif]

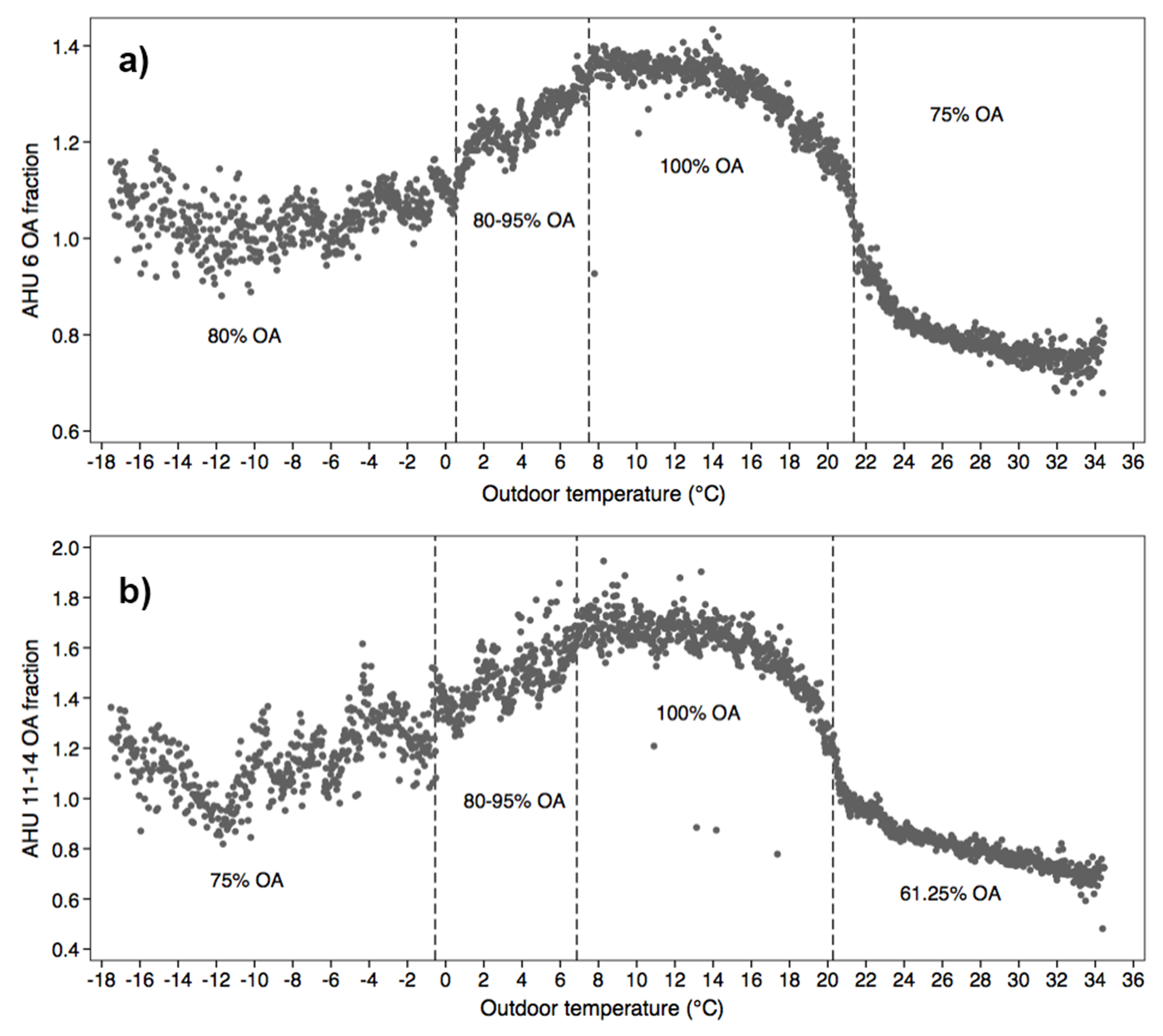

Supplement: S2 Fig — The HVAC systems operated in economizer mode, varying OA fraction with outdoor temperature conditions. (TIF) [file pone.0118207.s002.tif]

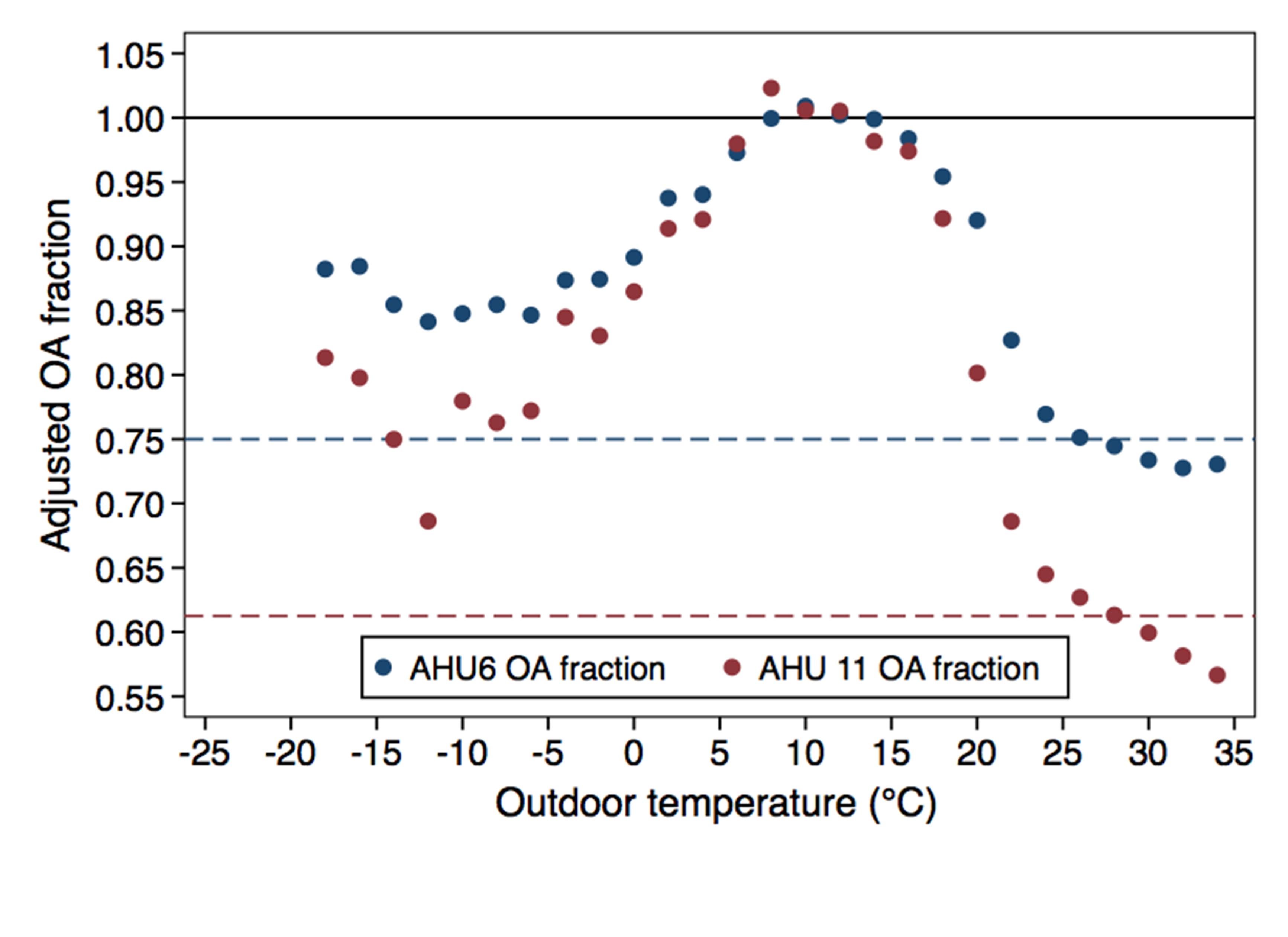

Supplement: S3 Fig — The HVAC systems operated in economizer mode, varying OA fraction with outdoor temperature conditions. (TIF) [file pone.0118207.s003.tif]

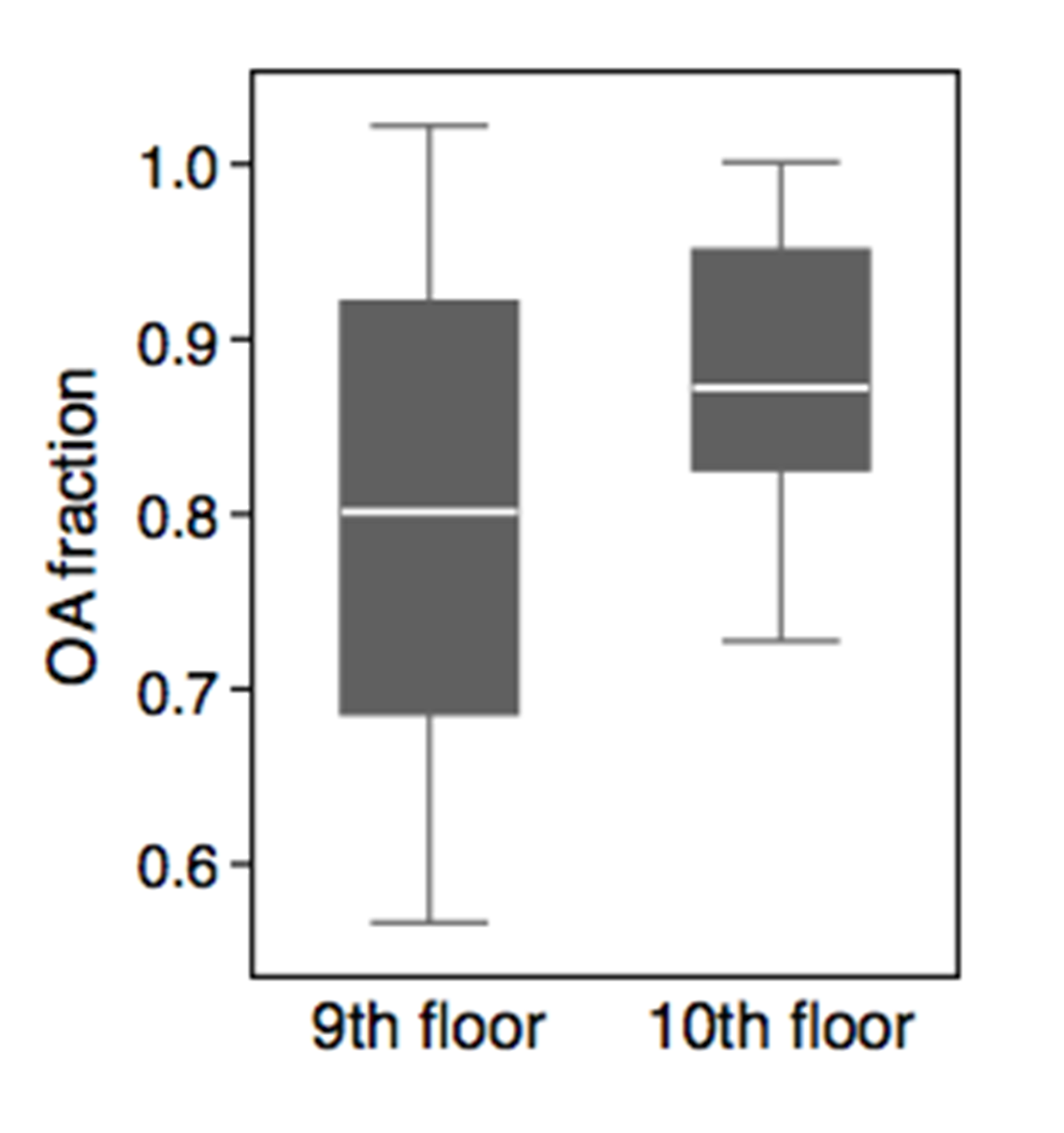

Supplement: S4 Fig — OA fractions in the HVAC systems were typically lower on the 9th floor than the 10th floor. (TIF) [file pone.0118207.s004.tif]

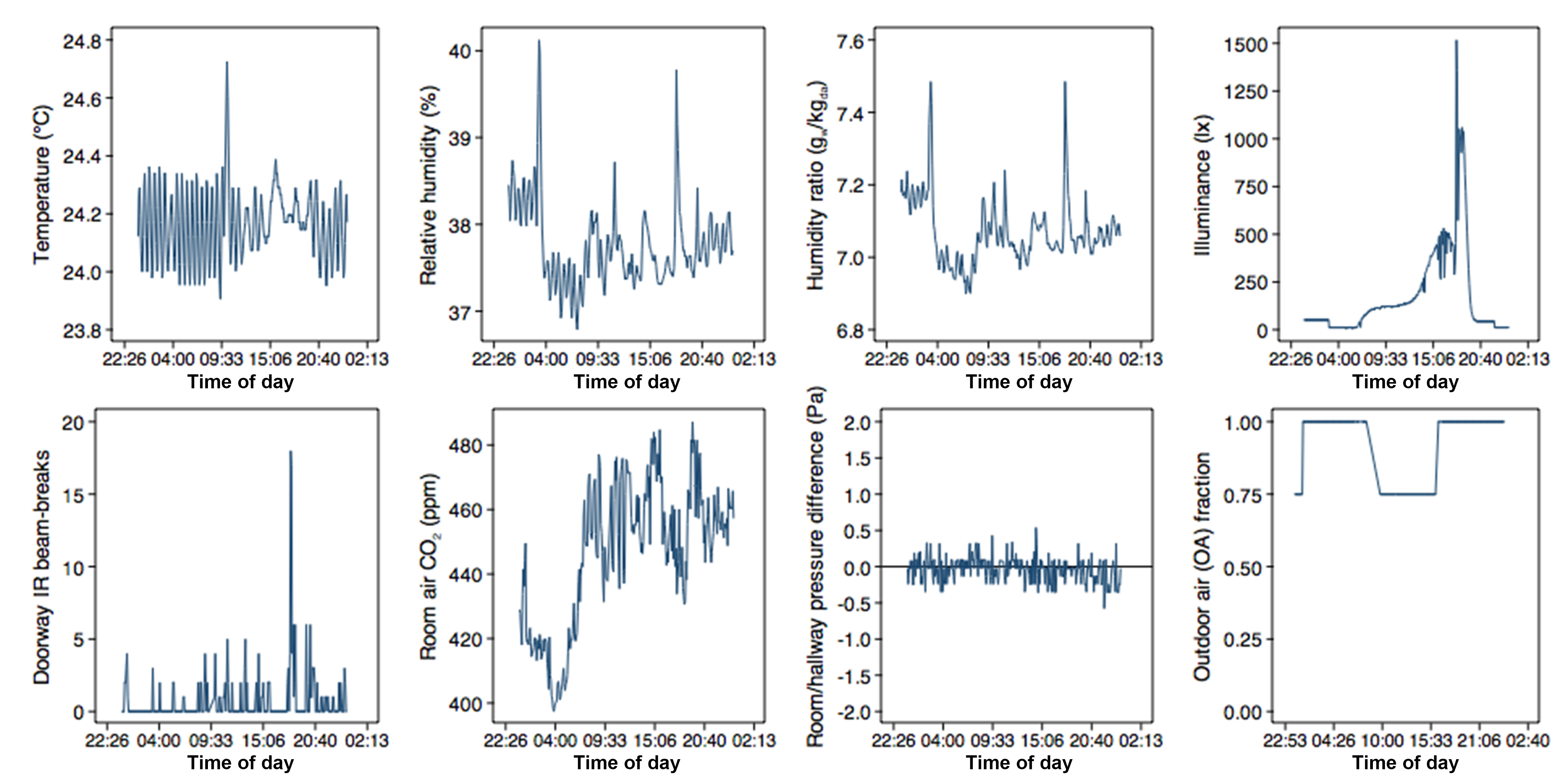

Supplement: S5 Fig — Example time series of 5-minute data for temperature, relative humidity, humidity ratio, illuminance, doorway IR beam-breaks, room air CO2 concentration, pressure difference between room and hallway, and HVAC outdoor air (OA) fraction for one room on one floor. (TIF) [file pone.0118207.s005.tif]

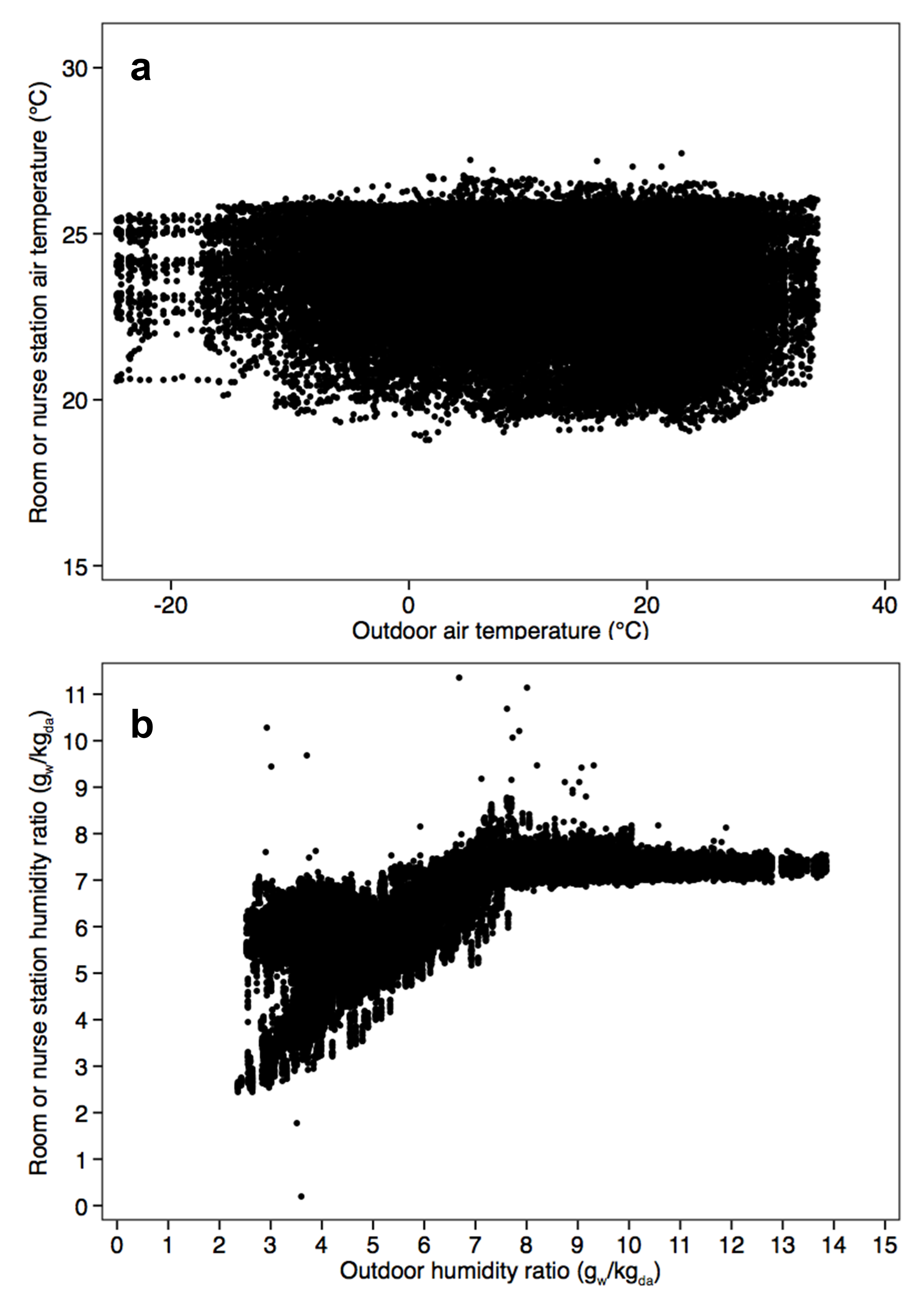

Supplement: S6 Fig — There was no correlation between indoor temperature and outdoor temperature. Indoor humidity ratio tracked outdoor humidity ratio until the outdoor humidity ratio reached ∼8 gw/kgda. (TIF) [file pone.0118207.s006.tif]

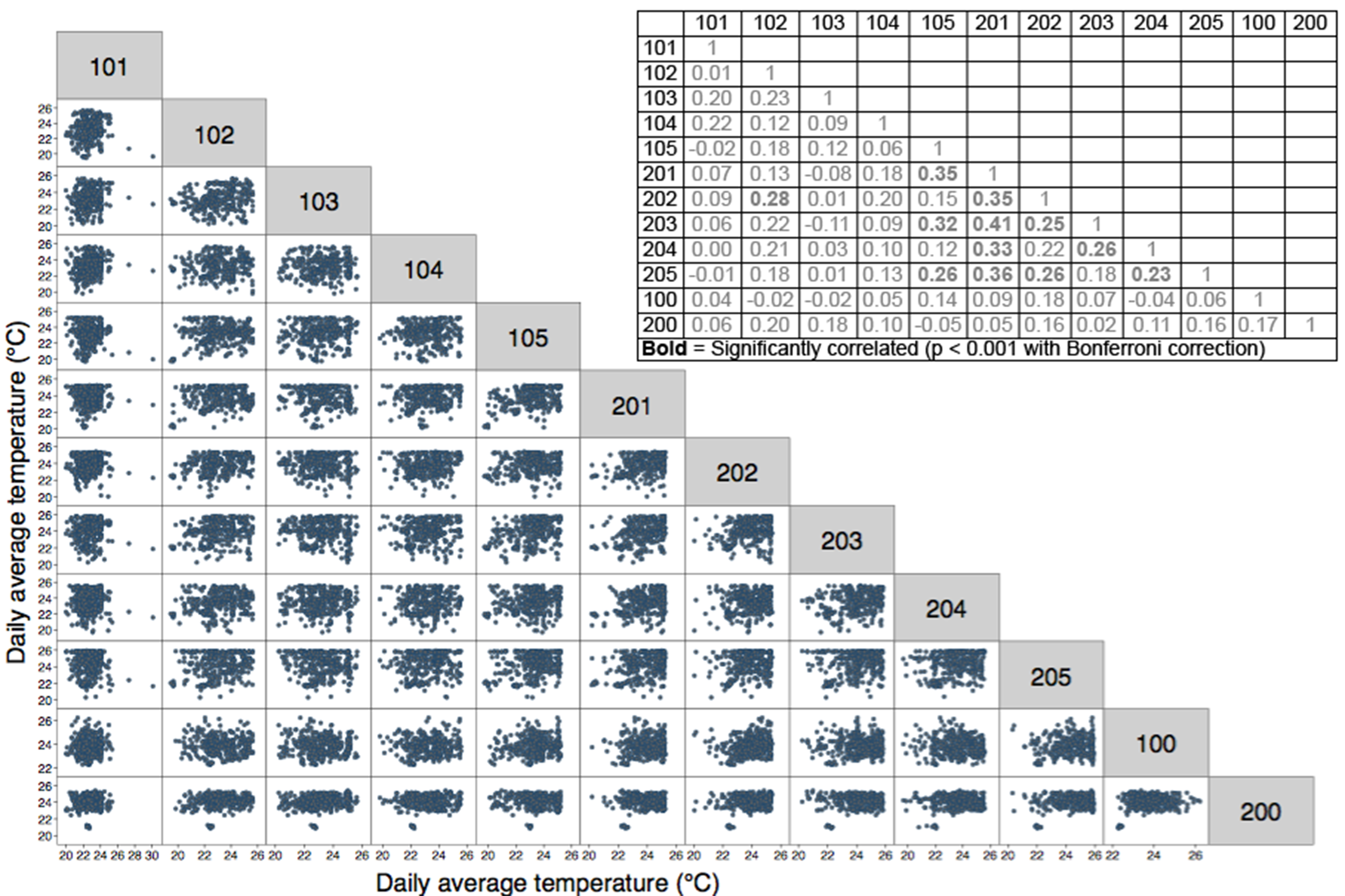

Supplement: S7 Fig — Daily average air temperatures were weakly correlated between locations. The inset table shows pair-wise Pearson correlation coefficients for each location comparison. (TIF) [file pone.0118207.s007.tif]

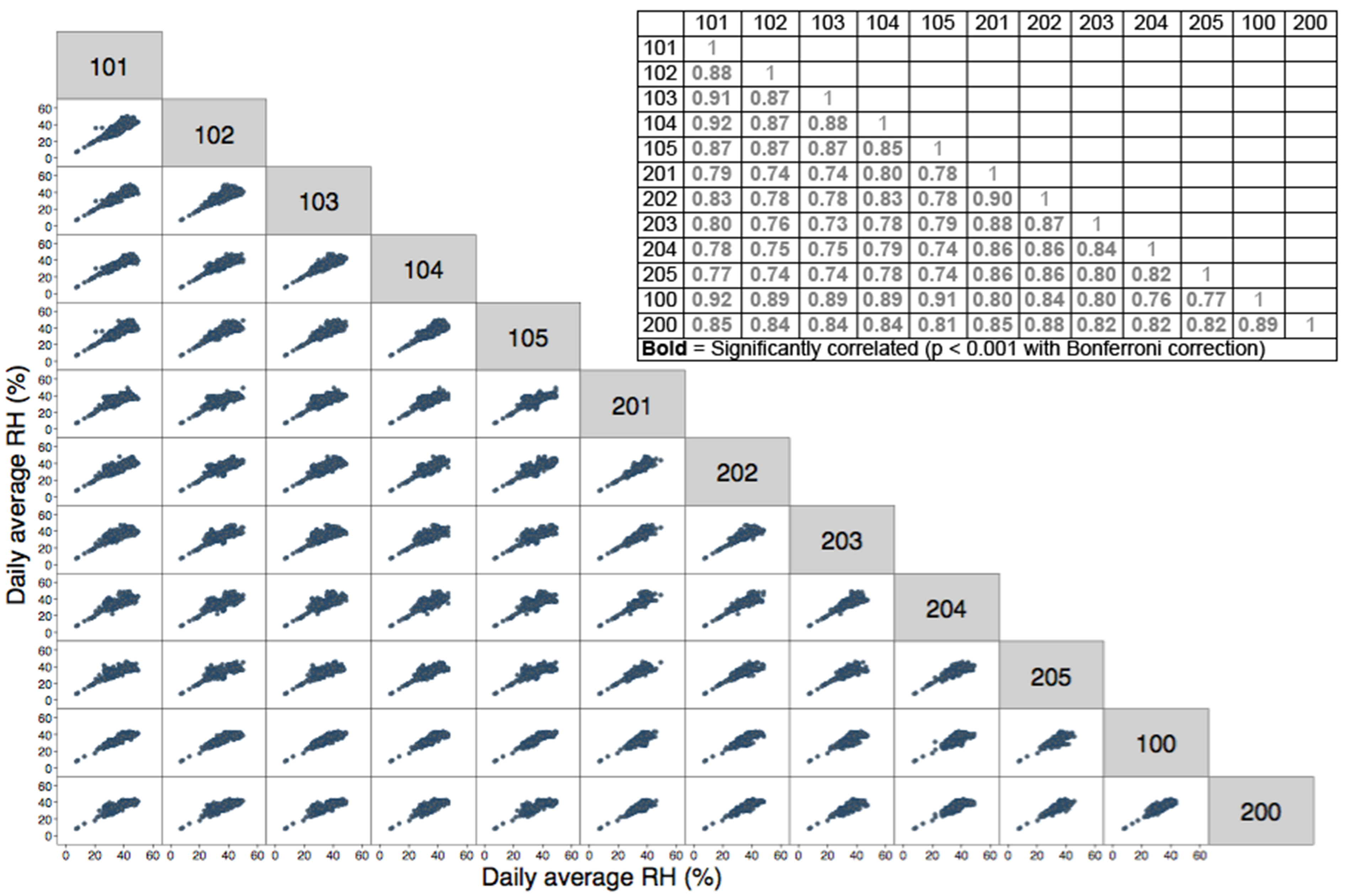

Supplement: S8 Fig — Daily average relative humidity measurements showed strong correlations between locations. The inset table shows pair-wise Pearson correlation coefficients for each location comparison. (TIF) [file pone.0118207.s008.tif]

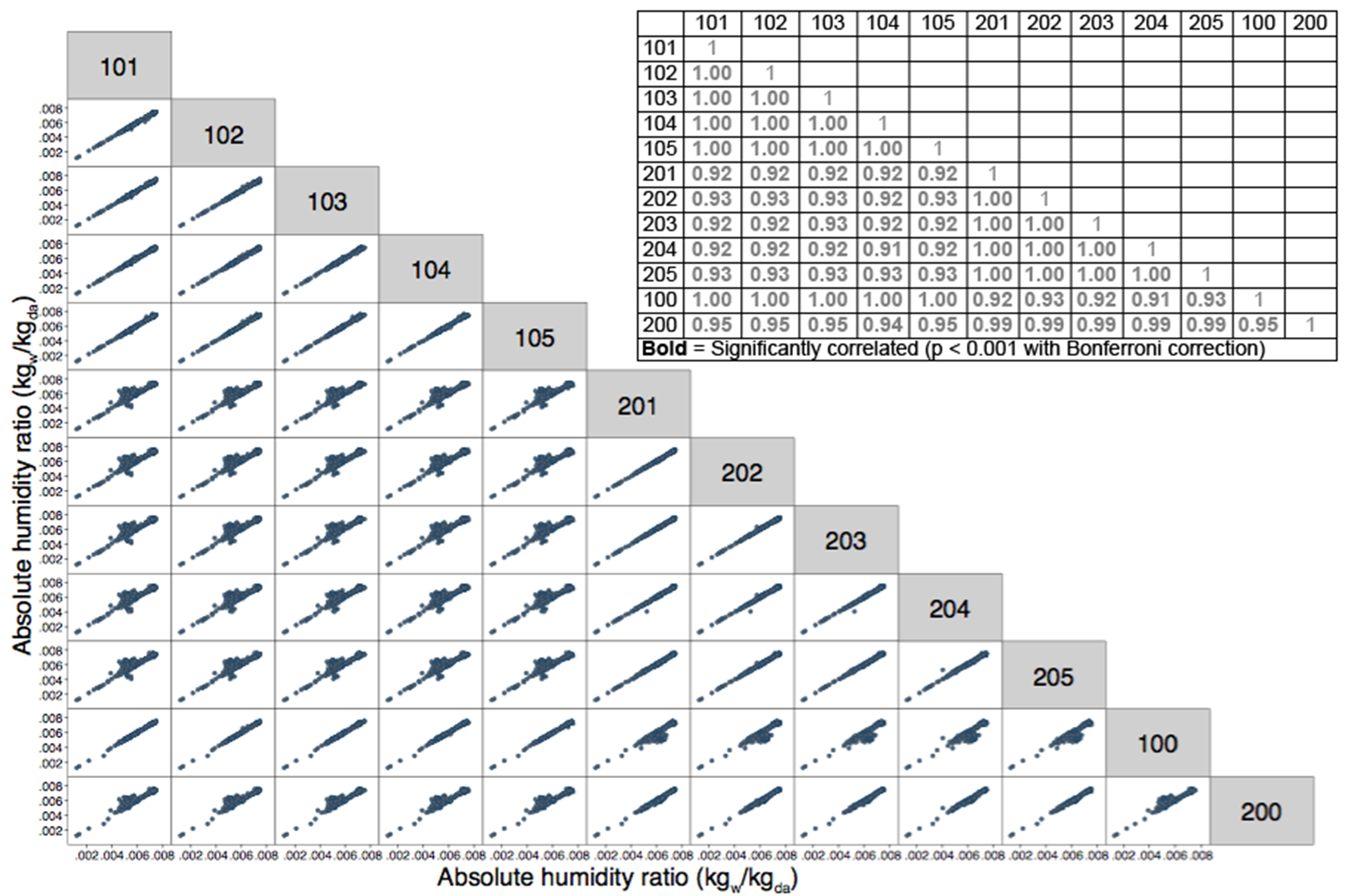

Supplement: S9 Fig — Daily average absolute humidity ratio measurements showed very strong correlations between locations. The inset table shows pair-wise Pearson correlation coefficients for each location comparison. (TIF) [file pone.0118207.s009.tif]

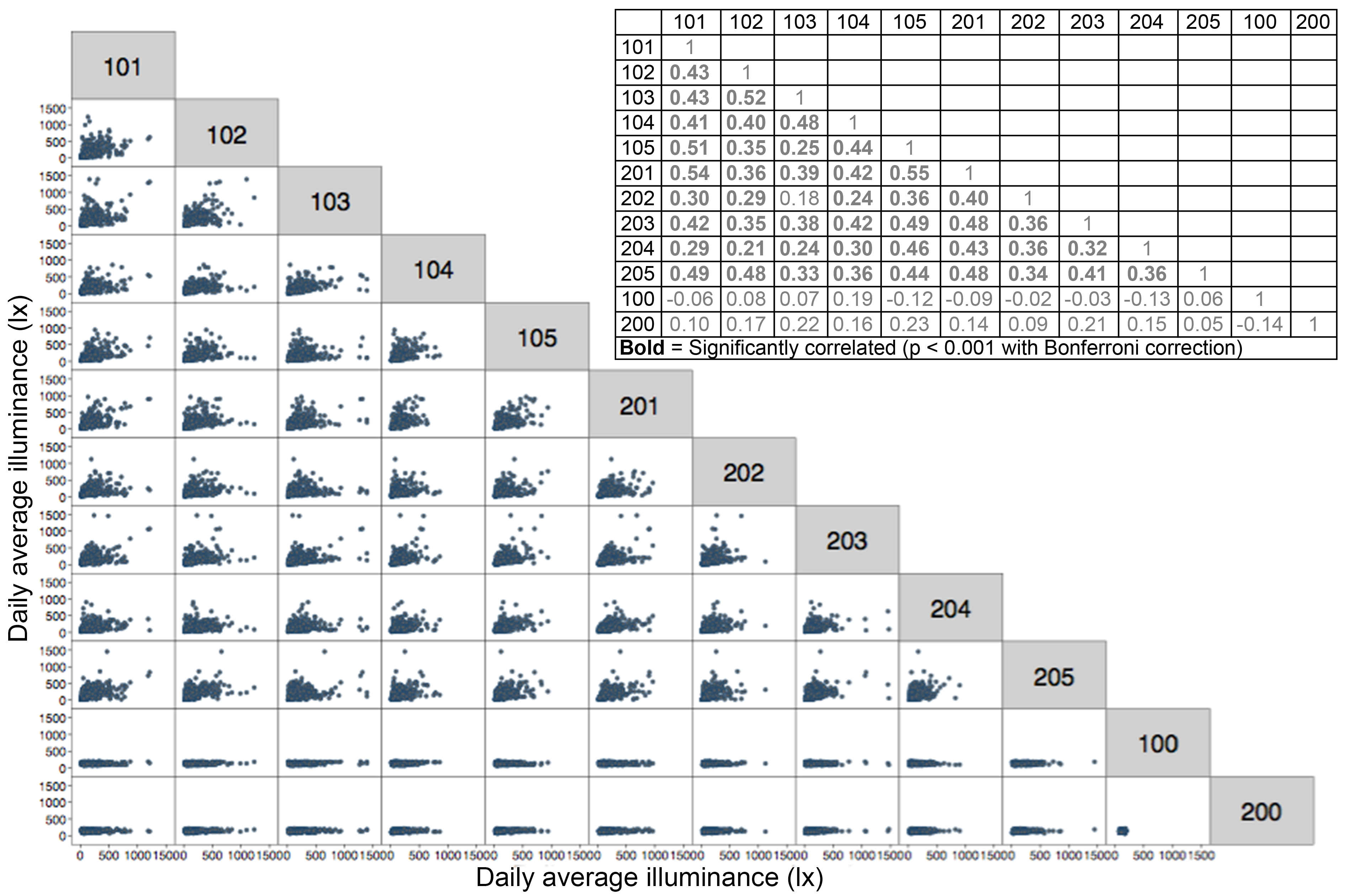

Supplement: S10 Fig — Daily average illuminance levels were moderately correlated between patient rooms, with no correlation between nurse stations. The inset table shows pair-wise Pearson correlation coefficients for each location comparison. (TIF) [file pone.0118207.s010.tif]

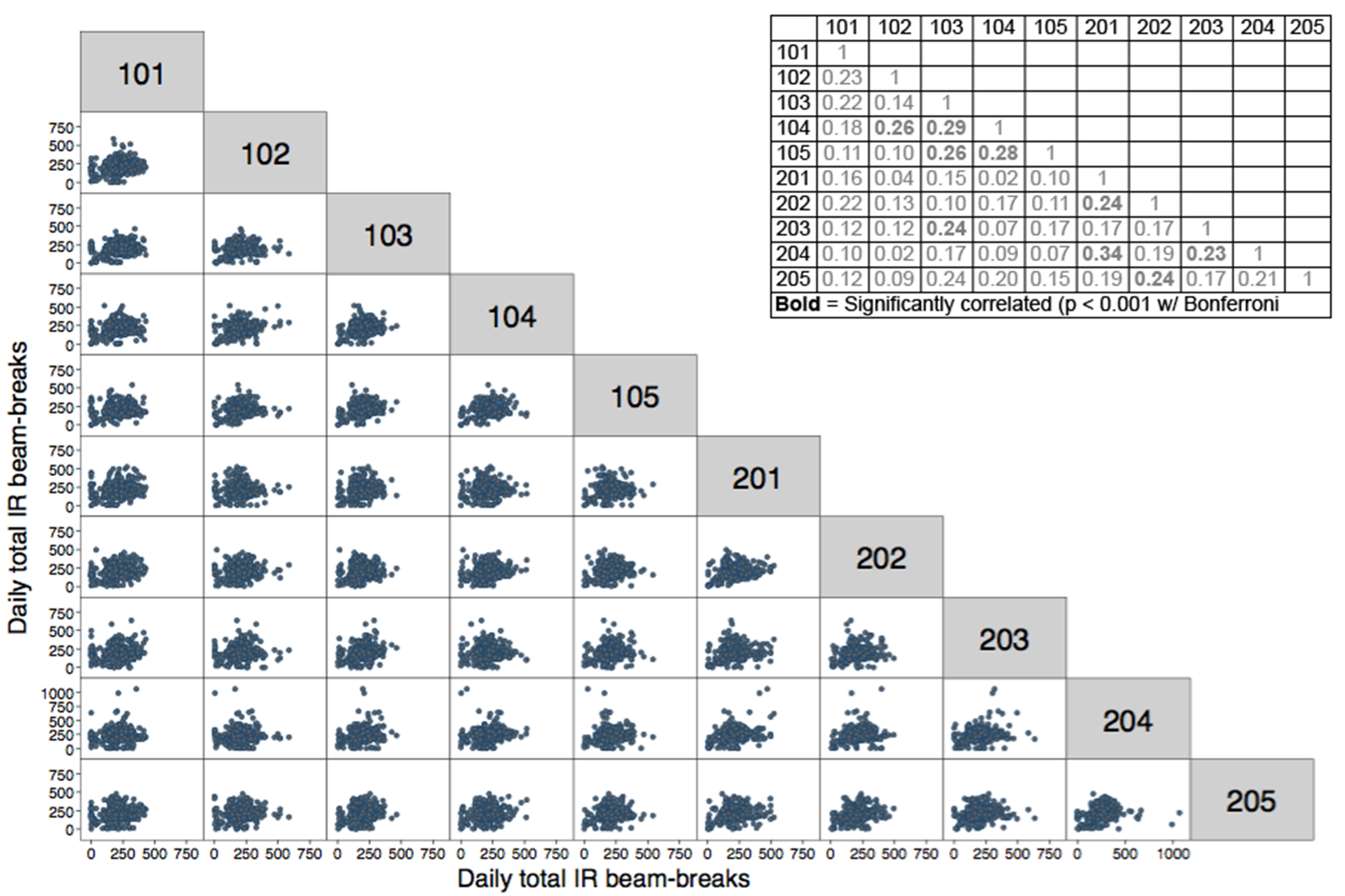

Supplement: S11 Fig — Daily total doorway IR beam-breaks showed little correlation between patient rooms. The inset table shows pair-wise Pearson correlation coefficients for each location comparison. (TIF) [file pone.0118207.s011.tif]

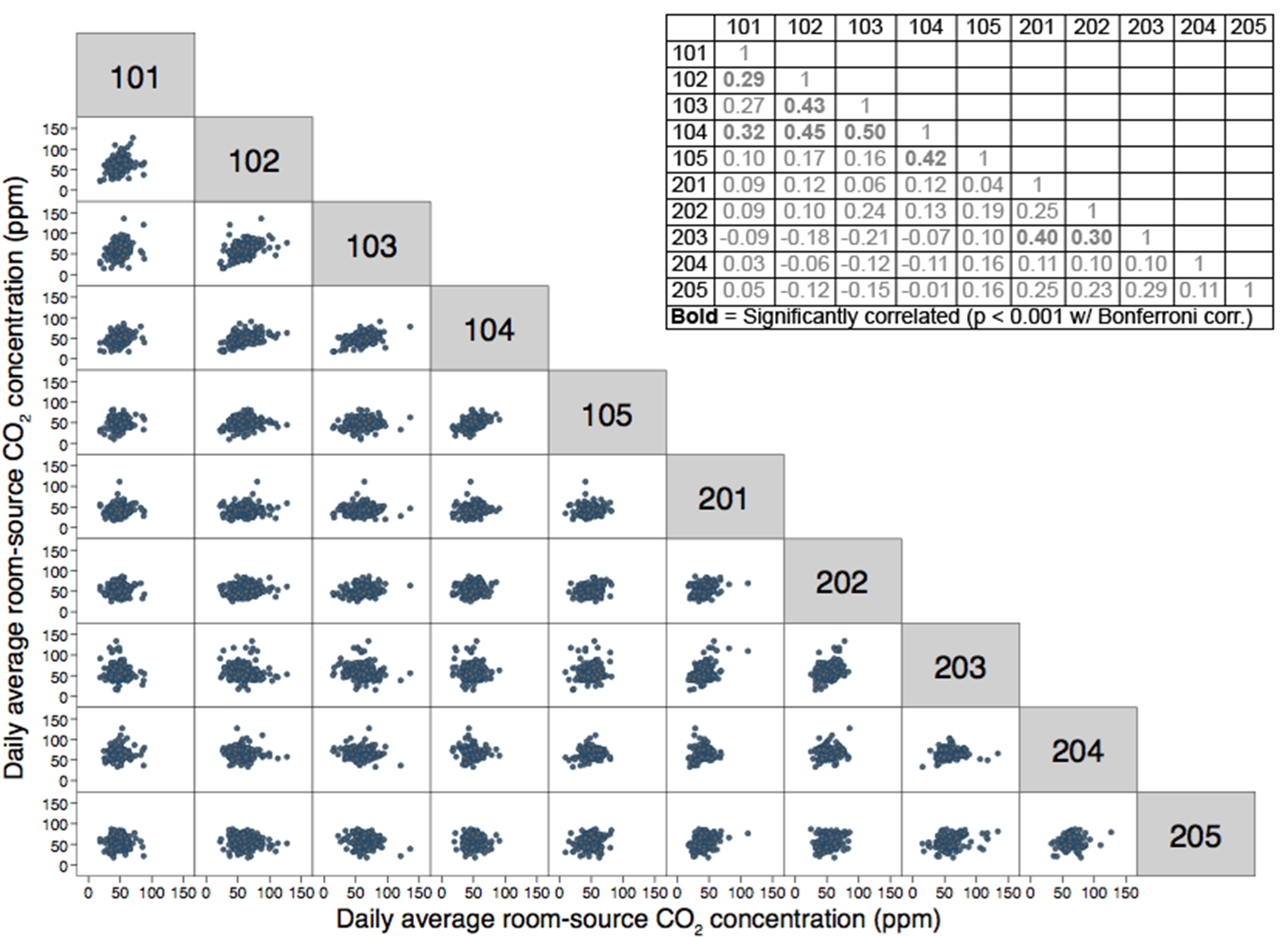

Supplement: S12 Fig — Daily average room-source CO2 concentrations showed little correlation between patient rooms. The inset table shows pair-wise Pearson correlation coefficients for each location comparison. (TIF) [file pone.0118207.s012.tif]
