# Supplementary material for: Spatial and Temporal Variations in Indoor Environmental Conditions, Human Occupancy, and Operational Characteristics in a New Hospital Building
Source: PLoS One. 2015 Mar 2;10(3):e0118207. doi: 10.1371/journal.pone.0118207 (PMC4346405; doi:10.1371/journal.pone.0118207)
Supplement: S1 File — This file also contains Tables A-D. Table A, Flow schedule and baseline measurements. Table B, Pair-wise correlation matrices for daily mean air temperature, relative humidity, humidity ratio, and illuminance levels in the patient rooms and nurse stations. Table C, Pair-wise correlation matrices for daily total IR beam-breaks and daily average room-source CO2 (and occupancy) in the patient rooms. Table D, Fractions of measured differences in daily mean temperature, relative humidity, and humidity ratio between patient rooms and nurse stations, as well as room-air CO2 concentrations in the patient rooms, that were within the range of propagated uncertainty. (DOC) [file pone.0118207.s013.doc]

# S1: Supporting Information

# Instrument Co-location, Initial Measurements, and QA/QC

This section describes initial instrument co-location experiments in the laboratory, initial measurements made in the hospital prior to occupation by patients and staff (including results from degree of mixing tests and airflow measurements), and QA/QC procedures for general sensor data collection and for estimating outdoor air fractions in the measured heating, ventilation, and air-conditioning (HVAC) systems.

## Instrument Co-Location

All 17 CO_2_ sensors used in the project were calibrated against one another using separate co-location experiments in a controlled laboratory before deployment. Calibration factors against a single CO_2_ sensor were estimated using linear regression and were applied to raw data retrieved from the sensors. This procedure was repeated approximately mid-way through the project with CO_2_ sensors from the mechanical room after some had failed there, and again in the laboratory at the conclusion of the project with all available CO_2_ sensors.

The 10 differential pressure sensors used in this work were also calibrated by co-location methods in the lab alongside an Energy Conservatory DG-700 differential pressure gauge prior to deployment. This was performed using a simple pressurized box connected to both the DG-700 and the pressure sensors to be launched in the field. Calibration factors were again estimated using linear regression and were applied to raw data after collection. Finally, an additional co-location calibration procedure was conducted in the laboratory with all of the temperature and relative humidity loggers used in the patient rooms and nurse stations at the close of the project.

Calibration factors appeared to have drifted considerably for most of the CO_2_ sensors over the course of the project. The final collected data set was analyzed using a combination of one or more of these calibration factors for each sensor, depending on how well it correlated with related data, particularly by comparing supply air CO_2_ measurements with patient room air CO_2_ measurements made during known unoccupied periods. These discrepancies introduce considerable uncertainty in our CO_2_ measurements. Conversely, the temperature and relative humidity sensor co-location experiment revealed that most of the sensors operated well within the range of uncertainty stated for each of the sensors (±0.4 °C for temperature, 2.5% for relative humidity). Using co-location calibration factors over the range of patient room temperatures measured herein (i.e., 17 °C to 31 °C), the temperature sensors appeared to be more accurate than suggested by the manufacturer-reported uncertainty: the mean temperature deviation was only 0.06 °C (ranging from 0.01 °C to 0.14 °C) and the mean relative humidity deviation was only 0.19% (ranging from 0.04% to 0.32%). Given these strong correlations with raw sensor data, calibration factors were not used for temperature and relative humidity data in this work. Illumination level and beam-break sensors were not used for co-location analyses.

## Degree of Mixing: CO_2_ Sensor Co-location Measurement

In order to test the extent of mixing inside the patient rooms, five calibrated CO_2_ sensors were installed in five separate locations within just one patient room (Room 102) and measured simultaneously for approximately 24 hours before the hospital opened (**S1 Fig.**). Measurement locations were chosen to cover a wide range of distances from each other within the relatively small patient room. Regression analysis showed that the room was reasonably well mixed throughout the duration of testing, even while unoccupied. CO_2_ measurements from each of the five locations were all within 5% of each other, including measurements taken under the counter in an exposed cabinet where the CO_2_ sensors were ultimately installed for long-term measurements.

## Airflow Measurements

During this same initial visit, baseline airflow rates were also measured in a number of the rooms. We used a pressure matching technique whereby a duct blaster was connected to a 0.6 m × 0.6 m × 0.6 m box and held against supply diffusers, return grilles, and bathroom exhaust grilles in most of the patient rooms. Results are shown in **Table A**. Flow measurements obtained from supply diffusers were difficult to obtain with high accuracy, due to the nature and shape of the supply diffusers. Therefore, we considered these values to be quite suspect compared to the more reliable return and exhaust flow measurements, as the measurement device fit over these grilles. Values in italics were found to be out of the range specified in the hospital’s airflow schedule. On the 9^th^ floor, both supply and return airflow measurements were found to be outside the range of the flow schedule. Return grille air flow measurements on the 9^th^ floor were shown to be consistently higher than supply air flow measurements, suggesting that additional flow was coming in from outside the patient room; however we have limited confidence in our supply flow measurement data because of difficulties described previously). The sum of the return and exhaust airflow rates were near 850 m^3^/hr in each patient room. Assuming the rooms were operated at neutral pressure, as specified in the mechanical plans and confirmed by measurements, supply airflow rates were likely closer to 850 m^3^/hr as well. For the patient room indoor air volume of ~97 m^3^, the rate of supply airflow to room volume (effectively the recirculation rate of the room) was approximately 8.8 per hour. Additionally, the volume of indoor air passing through the HVAC filter media passive air sampler on the return grill (which was installed/replaced on a weekly basis) was approximately 1.14×10^5^ m^3^. On the 10^th^ floor, with the exception of the last room (Room 205), only the return flow measurements were found to be outside the flow range identified in the schedule. In the last room, both supply and return flows were found to be outside of the ranges specified in the schedule. Most of the supply airflow rate measurements were found to be higher than those at the return grille, but these values are suspect due to potential errors in the measurement procedure. Additionally, there is no way of knowing whether airflow rates were adjusted between the time measurements were made and the time the hospital actually opened.

## HVAC System Outdoor Air Fraction QA/QC

Raw outdoor air fractions were often outside of the bounds of theoretical limits (0 to 1) because of uncertainties associated with very low differences in CO_2_ concentrations between the three airstreams in each air handling unit (AHU). However, we periodically retrieved short-term records of outdoor air damper positions from the facilities manager at the hospital for our measured AHUs, which allowed for periodic calibration of our absolute values against their data. Unfortunately their system only stored the previous 24-hours of data, so long-term calibration was not feasible. Additionally, because we measured temperature in the outdoor air stream and the hospital HVAC system operated with an economizer, we had another method of estimating outdoor air fractions based on outdoor temperature alone.

Both HVAC systems were shown to have a maximum raw outdoor air fraction occurring when outdoor air temperatures are between approximately 7 °C and 16 °C, which is consistent with typical economizer operation. Outdoor air dampers were set at 100% during most of these periods, which was determined by examining facilities data. We also know that minimum outdoor air damper settings were 75% in AHU 6; thus our minimum outdoor air fraction estimates made during the highest outdoor air temperatures likely correspond to 75% in AHU 6. AHU 11 (consisting of AHUs 11-14) was more complicated. AHU 11 and 12 had minimum damper settings of 50% each (according to the facilities department), while AHU 13 had a minimum of 75% and AHU 14 had a minimum of 70%. Because each of these AHUs had the same design airflow rate, we can reasonably assume that the minimum outdoor air fraction for the combination of these four AHUs was the average of the four individual minimum outdoor air fractions, which is approximately 61.25%.

Raw outdoor air fractions were averaged over a number of small temperature bins and plotted against the mean temperature in that temperature bin, revealing a tighter pattern as shown in **S2 Fig**. Using the known minimum damper positions of 75% and 61.25% for AHU 6 and AHU 11 respectively, we applied these to the lowest outdoor air fractions which occurred at higher temperatures, with a lower boundary of approximately 20 °C. We assumed that the highest outdoor air fraction is 100%, occurring at moderate temperatures between 7 °C and 20 °C. Below 7 °C, the outdoor air fraction appears to decrease linearly with temperature until about 0 °C, where it plateaus. To further simplify the analysis and calibrate the raw data to reveal adjusted outdoor air fractions, raw outdoor air fractions were averaged over 2 °C wide temperature bins and the minimum and maximum raw outdoor air fractions from these data, along with our known real outdoor air fractions, were used to calculate a calibration factor to obtain an estimates of adjusted outdoor air fractions over all temperatures.

**S3 Fig.** shows scaled outdoor air fractions for both AHU 6 and AHU 11 after applying calibration factors. For both AHUs, outdoor air fractions appear to drop below stated minimum outdoor air fractions. Based on our analysis of data received from the hospital facilities department, outdoor air fractions were at a minimum for all temperatures above ~21 °C, so calibration factors were determined such that all of these data points were minimum outdoor air fractions. Calibration factors were also scaled with the consideration that outdoor air fractions were between 80 and 90% for lowest temperatures, also based on data from the hospital facilities department.

These data reveal differences in the amount of outdoor air delivered to the two hospital floors examined in this study. In general, rooms on the 10^th^ floor (AHU 6) received larger fractions of outdoor air during periods of both warm and cold weather than rooms on the 9^th^ floor (AHU 11); although during moderate temperatures both units operated at 100% outdoor air fractions. These data are also shown in **S4 Fig**. These differences could help to explain some of the observed variations in environmental parameters between floors.

Combining these outdoor air fractions with estimates of supply airflow rates at neutral pressure (where supply airflow rates equal the sum of return and exhaust flow rates in the patient rooms), room air exchange rates are estimated to vary from as low as ~5.2 per hour (at 60% outdoor air) to as much as ~8.8 per hour (at 100% outdoor air). However, it is not clear how important these differences will be for airborne microbial communities because the patient rooms were still operated at neutral pressure throughout the project and HEPA filtration was used on the supply airstream, so supply air should be relatively free of indoor-generated biological particulate matter, regardless of outdoor air fraction.

## Data Loss

The temperature, relative humidity, and illuminance sensors had minimal issues throughout the project and resulted in very little data loss. However, there was a lower total number of 5-minute data points collected for IR beam-break counts, CO_2_ concentrations, and differential pressure measurements because of a number of sensor and calibration issues throughout the project. The issues included loss of battery power, corrupted data logger files, sensor failure, improper sensor placement (i.e., IR beam-break placement prior to July 3, 2013), or sensors temporarily removed and misplaced by hospital staff. Approximately 28% of the possible patient room CO_2_ data points were either not collected or excluded for QA/QC reasons (i.e., measurements were outside of reasonable bounds or sensors were pulled for calibration or maintenance). Approximately 21% of the possible IR beam-break data points were also lost or excluded due to sensor placement issues. The total number of data points for differential pressure was also much lower as these sensors were installed about 3 months after the beginning of our measurement period, and also account for data dropped due to sensor issues. Although the range of differential pressure measurements is between about -1.8 and 1.8 Pa, the mean remained close to zero, 0.09 Pa. The placement of these sensors was next to patient room doorways, which caused frequent interference and some fluctuations in pressure, but overall patterns indicate values hovered close to zero.

# Example Time-series Data (5-min intervals)

**S5 Fig.** shows a panel of example 24-hour time-series data from a number of different patient rooms (at 5-minute intervals), including temperature, relative humidity, humidity ratio, illuminance levels, doorway IR beam breaks, room air CO_2_ concentrations, differential pressure with respect to the hallway, and estimated outdoor air fractions in the AHU serving this space. These are meant to provide just one example of a 24-hour period out of the entire yearlong project, but are not representative of every day of data collection.

Air temperature (**S5a Fig.**) shows a pattern of oscillations around a set point, typical of HVAC cycling. Relative humidity (**S5b Fig.**) and humidity ratio (**S5c Fig.**) show similar profiles around narrow bands. Illuminance levels (**S5d Fig.**) show a characteristic pattern of a combination of solar radiation increasing throughout the day and low nighttime lighting (small step changes between around 8 pm and 5 am), peaking in the west-facing rooms around 5 or 6 pm. IR beam-breaks (**S5e Fig.**) show relatively low activity in late night and early morning hours, with higher intensity peaks around mid-morning and again in late afternoon or early evening. CO_2_ concentrations (**S5f Fig.**) on this example day were lowest in the middle of the night (likely attributed to periods of lower occupancy; lower occupant CO_2_ emission rates for typical nighttime activities, such as sleeping; or different ventilation schedules) and increased by morning, remaining relatively consistent throughout the rest of the day. Room pressure differential (**S5g Fig.**) shows a characteristic signal hovering around 0 Pa (generally within propagated instrument uncertainty). Finally, calibrated outdoor air fractions (**S5h Fig.**) show peaks of 100% OA during morning and night periods, with lower fractions around 75% during the day in response to higher temperatures. The next sections explore these same data, but averaged over more meaningful time-scales (i.e., hourly and daily), and use these data to explore variability within rooms, between rooms, and between floors.

# Full correlation matrices

**Table B** shows the full correlation matrices for daily mean air temperatures, relative humidity, humidity ratio, and illuminance levels between the patient rooms and nurse stations. **Table C** shows the full correlation matrices for daily total IR beam-breaks and daily average room-source CO_2_ concentrations (and average estimated occupancy based on room-source CO_2_) between the patient rooms. **Table D** shows the fraction of measured differences in daily mean temperature, relative humidity, and humidity ratio between patient rooms and nurse stations that were within the range of propagated uncertainty.

# Tables

**Table A. Flow schedule and baseline measurements.**

| Room | Measured (m^3^/hr)^*^ | | | Schedule (m^3^/hr) | | | |
| --- | --- | --- | --- | --- | --- | --- | --- |
|  | Supply | Return | Bathroom | Supply | | Return | |
|  |  |  |  | Max | Min | Max | Min |
| 101 | *632* | *700* | 170 | 765 | 663 | 595 | 493 |
| 102 | *578* | *680* | 170 | 765 | 663 | 595 | 493 |
| 103 | *586* | *680* | 170 | 765 | 663 | 595 | 493 |
| 104 | *586* | *646* | 170 | 765 | 663 | 595 | 493 |
| 105 | *635* | *637* | 170 | 765 | 663 | 595 | 493 |
| 201 | 681 | *646* | 170 | 765 | 663 | 595 | 493 |
| 202 | 698 | *646* | 170 | 765 | 663 | 595 | 493 |
| 203 | 671 | *663* | 170 | 765 | 663 | 595 | 493 |
| 204 | 680 | *629* | 170 | 765 | 663 | 595 | 493 |
| 205 | *646* | *688* | 170 | 765 | 663 | 595 | 493 |
| ^*^Italicized values may be suspect because they are out of the range described in the schedule | | | | | | | |

**Table B. Pair-wise correlation matrices for daily mean air temperature, relative humidity, humidity ratio, and illuminance levels in the patient rooms and nurse stations.**

| **Room #** | **101** | **102** | **103** | **104** | **105** | **201** | **202** | **203** | **204** | **205** | **100** | **200** |
| --- | --- | --- | --- | --- | --- | --- | --- | --- | --- | --- | --- | --- |
| **101** | 1 | **Air temperature** | | | | | | | | | | |
| **102** | 0.009 | 1 |  |  |  |  |  |  |  |  |  |  |
| **103** | 0.199 | 0.226 | 1 |  |  |  |  |  |  |  |  |  |
| **104** | 0.224 | 0.125 | 0.091 | 1 |  |  |  |  |  |  |  |  |
| **105** | -0.024 | 0.179 | 0.121 | 0.064 | 1 |  |  |  |  |  |  |  |
| **201** | 0.071 | 0.135 | -0.075 | 0.176 | 0.349* | 1 |  |  |  |  |  |  |
| **202** | 0.086 | 0.277* | 0.010 | 0.200 | 0.149 | 0.351* | 1 |  |  |  |  |  |
| **203** | 0.063 | 0.222 | -0.107 | 0.095 | 0.323* | 0.408* | 0.248* | 1 |  |  |  |  |
| **204** | -0.003 | 0.207 | 0.029 | 0.104 | 0.123 | 0.331* | 0.223 | 0.263* | 1 |  |  |  |
| **205** | -0.010 | 0.185 | 0.011 | 0.131 | 0.264* | 0.359* | 0.261* | 0.182 | 0.231* | 1 |  |  |
| **100** | 0.042 | -0.022 | -0.021 | 0.049 | 0.138 | 0.094 | 0.175 | 0.074 | -0.037 | 0.057 | 1 |  |
| **200** | 0.062 | 0.197 | 0.177 | 0.101 | -0.051 | 0.046 | 0.160 | 0.016 | 0.114 | 0.160 | 0.167 | 1 |
| **101** | 1 | **Relative humidity** | | | | | | | | | | |
| **102** | 0.882* | 1 |  |  |  |  |  |  |  |  |  |  |
| **103** | 0.910* | 0.873* | 1 |  |  |  |  |  |  |  |  |  |
| **104** | 0.915* | 0.874* | 0.883* | 1 |  |  |  |  |  |  |  |  |
| **105** | 0.869* | 0.866* | 0.866* | 0.855* | 1 |  |  |  |  |  |  |  |
| **201** | 0.789* | 0.738* | 0.744* | 0.796* | 0.782* | 1 |  |  |  |  |  |  |
| **202** | 0.828* | 0.783* | 0.782* | 0.827* | 0.776* | 0.898* | 1 |  |  |  |  |  |
| **203** | 0.802* | 0.758* | 0.734* | 0.784* | 0.787* | 0.884* | 0.868* | 1 |  |  |  |  |
| **204** | 0.784* | 0.755* | 0.747* | 0.785* | 0.736* | 0.861* | 0.862* | 0.835* | 1 |  |  |  |
| **205** | 0.773* | 0.738* | 0.740* | 0.775* | 0.742* | 0.856* | 0.859* | 0.802* | 0.820* | 1 |  |  |
| **100** | 0.919* | 0.890* | 0.892* | 0.890* | 0.905* | 0.798* | 0.838* | 0.797* | 0.762* | 0.769* | 1 |  |
| **200** | 0.854* | 0.843* | 0.845* | 0.837* | 0.805* | 0.854* | 0.883* | 0.821* | 0.823* | 0.822* | 0.887* | 1 |
| **101** | 1 | **Humidity ratio** | | | | | | | | | | |
| **102** | 0.998* | 1 |  |  |  |  |  |  |  |  |  |  |
| **103** | 0.998* | 0.998* | 1 |  |  |  |  |  |  |  |  |  |
| **104** | 0.998* | 0.997* | 0.997* | 1 |  |  |  |  |  |  |  |  |
| **105** | 0.998* | 0.998* | 0.997* | 0.997* | 1 |  |  |  |  |  |  |  |
| **201** | 0.923* | 0.920* | 0.924* | 0.919* | 0.923* | 1 |  |  |  |  |  |  |
| **202** | 0.927* | 0.926* | 0.929* | 0.923* | 0.928* | 0.999* | 1 |  |  |  |  |  |
| **203** | 0.924* | 0.922* | 0.926* | 0.920* | 0.924* | 0.999* | 0.998* | 1 |  |  |  |  |
| **204** | 0.919* | 0.917* | 0.921* | 0.915* | 0.919* | 0.996* | 0.996* | 0.996* | 1 |  |  |  |
| **205** | 0.929* | 0.928* | 0.931* | 0.925* | 0.930* | 0.998* | 0.998* | 0.998* | 0.995* | 1 |  |  |
| **100** | 0.998* | 0.998* | 0.998* | 0.997* | 0.998* | 0.919* | 0.926* | 0.922* | 0.914* | 0.929* | 1 |  |
| **200** | 0.950* | 0.947* | 0.949* | 0.945* | 0.949* | 0.988* | 0.991* | 0.989* | 0.986* | 0.992* | 0.953* | 1 |
|  | | | | | | | | | | | | |
| **101** | 1 | **Illuminance levels** | | | | | | | | | | |
| **102** | 0.435* | 1 |  |  |  |  |  |  |  |  |  |  |
| **103** | 0.427* | 0.524* | 1 |  |  |  |  |  |  |  |  |  |
| **104** | 0.406* | 0.402* | 0.478* | 1 |  |  |  |  |  |  |  |  |
| **105** | 0.513* | 0.349* | 0.253* | 0.438* | 1 |  |  |  |  |  |  |  |
| **201** | 0.544* | 0.356* | 0.393* | 0.419* | 0.549* | 1 |  |  |  |  |  |  |
| **202** | 0.305* | 0.292* | 0.176 | 0.238* | 0.360* | 0.397* | 1 |  |  |  |  |  |
| **203** | 0.424* | 0.347* | 0.375* | 0.423* | 0.490* | 0.484* | 0.358* | 1 |  |  |  |  |
| **204** | 0.293* | 0.214 | 0.242* | 0.295* | 0.461* | 0.431* | 0.360* | 0.319* | 1 |  |  |  |
| **205** | 0.491* | 0.478* | 0.328* | 0.357* | 0.436* | 0.479* | 0.345* | 0.410* | 0.362* | 1 |  |  |
| **100** | -0.060 | 0.079 | 0.074 | 0.188 | -0.122 | -0.089 | -0.018 | -0.029 | -0.134 | 0.061 | 1 |  |
| **200** | 0.098 | 0.175 | 0.217 | 0.165 | 0.229 | 0.144 | 0.089 | 0.215 | 0.149 | 0.051 | -0.139 | 1 |
| * Significantly correlated (p < 0.001 with Bonferroni correction) | | | | | | | | | | | | |

**Table C. Pair-wise correlation matrices for daily total IR beam-breaks and daily average room-source CO_2_ (and occupancy) in the patient rooms.**

| **Room #** | **101** | **102** | **103** | **104** | **105** | **201** | **202** | **203** | **204** | **205** |
| --- | --- | --- | --- | --- | --- | --- | --- | --- | --- | --- |
| **101** | 1 | **Daily total IR beam-breaks** | | | | | | | | |
| **102** | 0.229 | 1 |  |  |  |  |  |  |  |  |
| **103** | 0.216 | 0.144 | 1 |  |  |  |  |  |  |  |
| **104** | 0.184 | 0.264* | 0.289* | 1 |  |  |  |  |  |  |
| **105** | 0.113 | 0.097 | 0.257* | 0.275* | 1 |  |  |  |  |  |
| **201** | 0.162 | 0.045 | 0.153 | 0.020 | 0.096 | 1 |  |  |  |  |
| **202** | 0.223 | 0.129 | 0.100 | 0.166 | 0.114 | 0.243* | 1 |  |  |  |
| **203** | 0.123 | 0.116 | 0.242* | 0.067 | 0.175 | 0.173 | 0.166 | 1 |  |  |
| **204** | 0.104 | 0.024 | 0.168 | 0.090 | 0.074 | 0.345* | 0.195 | 0.229* | 1 |  |
| **205** | 0.123 | 0.086 | 0.239 | 0.203 | 0.147 | 0.192 | 0.237* | 0.174 | 0.214 | 1 |
| **101** | 1 | **Daily average room-source CO_2_ (& average est. occupancy)** | | | | | | | | |
| **102** | 0.294* | 1 |  |  |  |  |  |  |  |  |
| **103** | 0.271 | 0.432* | 1 |  |  |  |  |  |  |  |
| **104** | 0.323* | 0.446* | 0.501* | 1 |  |  |  |  |  |  |
| **105** | 0.104 | 0.172 | 0.158 | 0.420* | 1 |  |  |  |  |  |
| **201** | 0.085 | 0.122 | 0.059 | 0.115 | 0.043 | 1 |  |  |  |  |
| **202** | 0.088 | 0.104 | 0.241 | 0.131 | 0.191 | 0.252 | 1 |  |  |  |
| **203** | -0.090 | -0.179 | -0.209 | -0.071 | 0.098 | 0.396* | 0.304* | 1 |  |  |
| **204** | 0.030 | -0.058 | -0.119 | -0.110 | 0.162 | 0.110 | 0.103 | 0.101 | 1 |  |
| **205** | 0.045 | -0.119 | -0.152 | -0.007 | 0.159 | 0.249 | 0.227 | 0.287 | 0.111 | 1 |
| * Significantly correlated (p < 0.001 with Bonferroni correction) | | | | | | | | | | |

**Table D. Fractions of measured differences in daily mean temperature, relative humidity, and humidity ratio between patient rooms and nurse stations, as well as room-air CO_2_ concentrations in the patient rooms, that were within the range of propagated uncertainty.**

| **Room #** | **101** | **102** | **103** | **104** | **105** | **201** | **202** | **203** | **204** | **205** | **100** |
| --- | --- | --- | --- | --- | --- | --- | --- | --- | --- | --- | --- |
| **102** | 0.271 | **Air temperature** | | | | | | | | | |
| **103** | 0.301 | 0.312 |  |  |  |  |  |  |  |  |  |
| **104** | 0.361 | 0.257 | 0.355 |  |  |  |  |  |  |  |  |
| **105** | 0.281 | 0.341 | 0.315 | 0.263 |  |  |  |  |  |  |  |
| **201** | 0.242 | 0.263 | 0.221 | 0.298 | 0.272 |  |  |  |  |  |  |
| **202** | 0.276 | 0.265 | 0.281 | 0.303 | 0.295 | 0.391 |  |  |  |  |  |
| **203** | 0.238 | 0.290 | 0.255 | 0.276 | 0.224 | 0.325 | 0.354 |  |  |  |  |
| **204** | 0.238 | 0.281 | 0.275 | 0.256 | 0.275 | 0.319 | 0.320 | 0.317 |  |  |  |
| **205** | 0.181 | 0.284 | 0.210 | 0.215 | 0.221 | 0.255 | 0.306 | 0.334 | 0.300 |  |  |
| **100** | 0.268 | 0.265 | 0.307 | 0.279 | 0.321 | 0.353 | 0.387 | 0.315 | 0.303 | 0.306 |  |
| **200** | 0.199 | 0.202 | 0.256 | 0.266 | 0.318 | 0.266 | 0.342 | 0.273 | 0.288 | 0.300 | 0.429 |
| **102** | 0.700 | **Relative humidity** | | | | | | | | | |
| **103** | 0.727 | 0.721 |  |  |  |  |  |  |  |  |  |
| **104** | 0.772 | 0.676 | 0.728 |  |  |  |  |  |  |  |  |
| **105** | 0.699 | 0.676 | 0.694 | 0.694 |  |  |  |  |  |  |  |
| **201** | 0.558 | 0.509 | 0.567 | 0.630 | 0.618 |  |  |  |  |  |  |
| **202** | 0.602 | 0.538 | 0.585 | 0.662 | 0.627 | 0.830 |  |  |  |  |  |
| **203** | 0.558 | 0.521 | 0.555 | 0.632 | 0.609 | 0.818 | 0.802 |  |  |  |  |
| **204** | 0.598 | 0.533 | 0.533 | 0.591 | 0.581 | 0.763 | 0.745 | 0.695 |  |  |  |
| **205** | 0.544 | 0.488 | 0.544 | 0.600 | 0.567 | 0.751 | 0.725 | 0.718 | 0.683 |  |  |
| **100** | 0.690 | 0.735 | 0.756 | 0.810 | 0.792 | 0.737 | 0.735 | 0.715 | 0.612 | 0.694 |  |
| **200** | 0.568 | 0.568 | 0.690 | 0.666 | 0.685 | 0.792 | 0.789 | 0.758 | 0.721 | 0.755 | 0.845 |
| **102** | 0.947 | **Humidity ratio** | | | | | | | | | |
| **103** | 0.894 | 0.829 |  |  |  |  |  |  |  |  |  |
| **104** | 0.945 | 0.896 | 0.841 |  |  |  |  |  |  |  |  |
| **105** | 0.897 | 0.959 | 0.643 | 0.858 |  |  |  |  |  |  |  |
| **201** | 0.504 | 0.481 | 0.382 | 0.435 | 0.504 |  |  |  |  |  |  |
| **202** | 0.401 | 0.462 | 0.276 | 0.361 | 0.476 | 0.970 |  |  |  |  |  |
| **203** | 0.419 | 0.464 | 0.309 | 0.356 | 0.507 | 0.973 | 0.975 |  |  |  |  |
| **204** | 0.462 | 0.467 | 0.377 | 0.438 | 0.476 | 0.960 | 0.890 | 0.925 |  |  |  |
| **205** | 0.340 | 0.431 | 0.244 | 0.326 | 0.476 | 0.897 | 0.946 | 0.948 | 0.801 |  |  |
| **100** | 0.940 | 0.981 | 0.714 | 0.932 | 0.976 | 0.526 | 0.443 | 0.488 | 0.503 | 0.421 |  |
| **200** | 0.568 | 0.603 | 0.390 | 0.477 | 0.613 | 0.673 | 0.631 | 0.639 | 0.682 | 0.609 | 0.643 |
| **102** | 0.953 | **Room-source CO_2_** | | | | | | | | | |
| **103** | 0.962 | 0.962 |  |  |  |  |  |  |  |  |  |
| **104** | 0.990 | 0.945 | 0.966 |  |  |  |  |  |  |  |  |
| **105** | 0.983 | 0.916 | 0.912 | 0.966 |  |  |  |  |  |  |  |
| **201** | 0.981 | 0.873 | 0.871 | 0.912 | 0.983 |  |  |  |  |  |  |
| **202** | 0.985 | 0.956 | 0.972 | 0.871 | 0.988 | 0.966 |  |  |  |  |  |
| **203** | 0.928 | 0.898 | 0.867 | 0.972 | 0.935 | 0.934 | 0.952 |  |  |  |  |
| **204** | 0.902 | 0.927 | 0.898 | 0.867 | 0.923 | 0.872 | 0.924 | 0.952 |  |  |  |
| **205** | 0.961 | 0.933 | 0.948 | 0.898 | 0.974 | 0.967 | 0.965 | 0.951 | 0.939 |  |  |
